# Medicinal Cannabis for Inflammatory Bowel Disease: A Survey of Perspectives, Experiences, and Current Use in Australian Patients

**DOI:** 10.1093/crocol/otaa015

**Published:** 2020-04-16

**Authors:** Melissa J Benson, Sarah V Abelev, Susan J Connor, Crispin J Corte, Lewis J Martin, Lucy K Gold, Anastasia S Suraev, Iain S McGregor

**Affiliations:** 1 Lambert Initiative for Cannabinoid Therapeutics, The University of Sydney, Sydney, New South Wales, Australia; 2 Faculty of Science, School of Psychology, The University of Sydney, Sydney, New South Wales, Australia; 3 The University of Sydney, Brain and Mind Centre, Sydney, New South Wales, Australia; 4 Department of Gastroenterology, Liverpool Hospital, Sydney, New South Wales, Australia; 5 Ingham Institute of Applied Medical Research, Liverpool, New South Wales, Australia; 6 The University of New South Wales, Sydney, New South Wales, Australia; 7 Department of Gastroenterology, Royal Prince Alfred Hospital, Sydney, New South Wales, Australia; 8 Faculty of Medicine and Health, Central Clinical School, The University of Sydney, Sydney, New South Wales, Australia

**Keywords:** cannabis, inflammatory bowel disease, survey

## Abstract

**Background:**

Medicinal cannabis (MC) is an increasingly utilized treatment option for various refractory diseases. While robust clinical evidence supporting MC efficacy in inflammatory bowel disease (IBD) is lacking, many IBD patients report using MC to obtain symptomatic relief. Understanding this use and associated outcomes may help inform future clinical trials.

**Methods:**

A cross-sectional anonymous online survey was conducted involving Australians with IBD. It examined attitudes and experiences with MC in relation to IBD management. The survey included validated sub-questionnaires assessing quality of life, medication adherence, IBD severity, and functional impairment.

**Results:**

A total of 838 responses were obtained. Results showed 25.3% (n = 212) of respondents were current or previous users of MC (18.1% current, 7.2% previous). Half of the current users also consumed cannabis recreationally although less frequently than for medicinal purposes. Cannabis consumption was via smoking (joints 34.2%; water pipe/bongs 14.5%) or as an oral liquid (19.7%) with products obtained from recreational dealers (44.6%), friends/family (26.1%), or self-grown (9.8%). Only 3 respondents reported using legally accessed products. Clinical ratings of IBD severity did not differ according to cannabis use although users reported more hospitalizations, less engagement with specialist services, and lower medication adherence. IBD symptoms reported as positively affected by cannabis included abdominal pain, stress, sleep, cramping, and anxiety. Most users (92.7%) endorsed cannabis as effective in symptom management. Cannabis-using ulcerative colitis patients reported better quality of life than nonusers on some measures.

**Conclusion:**

Many patients in Australia are using illicit MC to manage their IBD. Further clinical trials are required to validate, or refute, patient claims around MC efficacy for symptom control in IBD.

## INTRODUCTION

The incidence of inflammatory bowel disease (IBD) continues to rise in Western countries^1^ and while many symptoms can be managed with medical therapy, there is consensus that IBD is not readily curable with available therapeutic strategies.^2^ The high symptom burden of IBD is responsible for enormous costs in terms of quality of life (QoL), with depression and anxiety common comorbidities in patients,^3^ as well as associated occupational, social, and economic impact.

As the current therapeutic options frequently leave patients with imperfect symptom control, IBD patients often experience a difficult trajectory and frequently seek alternative treatment options to manage their symptoms.^4^ Recently, medicinal cannabis (MC) has emerged as a potential therapeutic in many areas of refractory disease^5^ and is being used experimentally by many patient cohorts, often in the absence of supportive clinical evidence.

The *Cannabis sativa* plant contains many bioactive molecules, the best characterized of which are ∆ ^9^-tetrahydrocannabiol (THC) and cannabidiol (CBD). Medicinal cannabis products are usually in the form of botanical material that is smoked or vaporised, or orally consumed extracts (oils). These typically contain THC, CBD, or a combination of the 2 as their core ingredients. Synthetic THC (dronabinol) and a chemically related analog (nabilone) are approved in some countries for use in chemotherapy-induced nausea and vomiting and AIDS-associated anorexia, while a 1:1 mixture of plant-derived THC and CBD (nabiximols) is approved for treating spasticity in multiple sclerosis.^6^ The therapeutic actions of THC are primarily due to an agonist action on cannabinoid type-1 receptors (CB1Rs)^7^ but this action also causes well-recognized side effects including sedation, appetite stimulation, and psychomotor-impairment. Unlike THC, CBD has no intoxicating effects and is well tolerated at high doses,^8^ reflecting an absence of agonist activity at the CB1R. CBD has recently gained FDA approval for use in Dravet syndrome and Lennox–Gastaut syndrome, both treatment-refractory pediatric epilepsies.^9, 10^ There are numerous clinical trials of CBD underway for many other conditions including anxiety, chronic pain, and addictive disorders.

The increased interest in the use of MC products for treating IBD reflects the more widespread community interest in MC products that is linked to their increased legal availability in many countries. It is also predicated on observed endocannabinoid influences on gastrointestinal function^11^ and encouraging findings of anti-inflammatory and wound healing effects of plant-derived cannabinoids in animal and cellular models of IBD.^12^

Clinical trials of MC products, however, have produced only mixed outcomes to date.^13–16^ In a small pilot trial, twice daily consumption of THC-containing cannabis cigarettes over 8 weeks did not significantly affect remission rates in patients with Crohn’s disease (CD) relative to placebo, although improvements in steroid dependency, appetite, and sleep were observed.^14^ In another trial, very low doses of CBD (20 mg/day oral, 8 weeks) were well tolerated but did not significantly alter disease markers or rates of remission in CD.^15^

In ulcerative colitis (UC), twice daily oral dosing of higher doses of CBD (300–500 mg/day) together with THC (14–23 mg/day) did not affect rates of clinical remission, although improvements in partial Mayo score and subjective global impression of disease activity were observed.^13^ THC-related adverse events resulted in low compliance in this study, reducing trial medication adherence in 41% of participants randomized to the active arm.

A smaller, as yet unpublished study,^16^ found that THC (23 mg/day inhaled) did not improve disease markers or remission rates in UC patients, but did improve clinical response as measured by disease activity index score. Finally, a recently conducted observational study^17^ involving prescribed MC products (predominantly raw cannabis plant material and THC:CBD containing products) showed reduced disease severity (as measured by the Harvey-Bradshaw Index) following a longitudinal assessment 12 months postinitiation of MC treatment.

These ambivalent clinical trial outcomes could reflect an intrinsic lack of cannabinoid efficacy or other factors such as choice of cannabinoid product, dosing regimen, route of administration, and lack of statistical power. In any case, such results do not seem to deter patient motivations to utilize MC. For example, a large (n > 1300) recent survey of the Australian population of (mostly illicit) MC users found 12.4% were self-medicating for gastrointestinal conditions such as IBD.^18^ Despite this, the relatively strict evidence-based access pathways to obtaining prescription MC in Australia means that there have only been 28 official approvals for IBD management out of more than 14,300 approvals at the time of writing (TGA, Freedom of Information request #1311).

With Australia having one of the highest international incidence rates of IBD (29.6 per 100,000^19^), we developed a specific survey of Australian IBD patients examining their perspectives and experiences with MC use. This included exploring the demographic and clinical characteristics of MC users versus nonusers, their experience with MC products, and perspectives on regulatory and legal issues, as well as identifying factors predicting cannabis use and symptomatic improvement in this population.

The current survey follows a number of smaller published surveys (n = 24–55 respondents) of cannabis use in IBD from the United States and Canada.^20–24^ Overall, these surveys found self-reported improvements in pain, diarrhea, and poor appetite with MC and identified predictors of MC use (ie, younger age, chronic pain/analgesic use, use of other complementary/alternative therapies, low health-related QoL, history of IBD surgery). Here we built upon these existing surveys by accessing a much larger patient cohort that could be subdivided into different IBD types [UC, CD, IBD unspecified (IBDU)] and also to allow systematic comparisons between users and nonusers of cannabis.

## METHODS

### Study Design

This was an anonymous cross-sectional online survey enrolling a self-selected sample of Australian adults who reported a confirmed diagnosis of IBD (CD, UC, or IBDU). The survey was hosted by the University of Sydney’s Research Electronic Data Capture (REDCap) system and ran between April 17 and June 19, 2019. Eligible participants were aged at least 18 years, received healthcare in Australia, and provided voluntary informed consent. There was no requirement for participants to currently use, have previously used, or considered using MC. Participants completed the survey only once.

### Questionnaire

An 82-item survey was produced that incorporated original questions and a number of existing validated instruments. The survey consisted of the following items:

(a) Original questions (20 items): these addressed demographics, IBD history/diagnosis, current clinical care (GP, specialist, pharmaceutical use), hospitalizations, satisfaction with current treatments/symptom control, and use of alternative therapies.
(b) Parts of previous consumer surveys by our group assessing Australians’ attitudes toward, and knowledge of, MC (26 items)^25^.
(c) *Short Inflammatory Bowel Disease Questionnaire (SIBDQ)*^26^: A 10-item IBD-specific QoL tool measuring the physical, emotional, and social effects of IBD during the past 2 weeks. A higher SIBDQ score indicates better QoL and daily function. SIBDQ scores range from 10 to 70.
(d) *EuroQoL Five Dimension Five Level Questionnaire (EQ-5D-5L)*^27^: A 6-item QoL tool spanning 5 dimensions of mobility, self-care, usual activities, pain/discomfort, and anxiety/depression, focusing on health today. Utility scores were calculated using Australian coefficients.^28^
(e) *Work Productivity and Activity Impairment Questionnaire (WPAI)*^29^: A 6-item tool used to measure the effect of a specific health problem (eg, IBD) on paid work and overall impairment of activity during the past week.
(f) *Medication Adherence Rating Scale (MARS)*^30^: A 10-item questionnaire used to measure patient adherence to their prescribed IBD medication during the past week. A MARS score of 0–10 was calculated with 10 indicating the best possible medication adherence.
(g) Short modified questionnaire specific to CD (Crohn’s Disease Activity Index; 4 items).
(h) Short modified questionnaire specific to UC (Mayo Index; 3 items).
(i) Short modified questionnaire specific to IBDU (Disease Activity Index; 1 item).

Items (g), (h), and (i) were included to explore disease severity in respondents. Given the survey was administered online and not in a clinical setting, these items were modified to omit components that would normally be assessed by a physician. The modified disease indices are described in Supplementary Table S2.

Current and previous cannabis users were asked more questions (n = 82 and n = 79 questions, respectively) than nonusers (n = 67 questions), and this also varied slightly depending on IBD type. The additional questions enquired about IBD symptoms for which cannabis was used, as well as perceived efficacy, side effects, preparation type, source, and frequency of cannabis use. All participants were asked for their opinions on regulatory issues relating to MC access in Australia. Gastroenterologists specializing in the treatment of IBD provided expert input and a review of the survey questions.

Participants were recruited through advertisements on Australian IBD consumer networks (Crohn’s and Colitis Australia, Bowel Cancer Australia, and The Gut Foundation), University websites, social media platforms (including Twitter and Facebook), and by word-of-mouth (see Supplementary Table S1*for a breakdown of recruitment sources*). MC online discussion groups and forums were not included in our direct recruitment strategy to limit bias in data collection and patient sampling. There was no reimbursement or financial incentive for taking part in the voluntary survey. At the end of the survey respondents were asked if they would like to be contacted for future clinical trials. If agreeable, they were provided a secure nonlinked platform to enter their contact details. Examples of the advertisements used to invite survey respondents (via IBD patient networks) are provided as Supplementary Materials.

### Statistical Analyses

Data were analyzed using SPSS 24 (IBM, USA), GraphPad Prism v7.04 (GraphPad, CA), and Python v3.7 software. Descriptive statistics were used to calculate means and standard deviations; Student’s *t*-tests and one- or two-way ANOVAs were used to compare continuous variables; and × ^2^ tests to compare categorical variables. Where n < 20, Fisher’s exact test was used to compare categorical variables.

The survey data were also analyzed for correlations between responses to specific questions (ie, *Are you under the care of a specialist?* or *Do you take pharmaceuticals to manage your IBD?*) and cannabis use and associated therapeutic benefits of cannabis use. This was achieved by calculating odds ratios with 95% confidence intervals. This assumed a log-normal distribution of the odds ratio, with statistical significance calculated using the × ^2^ test for independence. These calculations were completed using the *statsmodels* Python library.^31^ A *P-*value of <0.05 was considered statistically significant.

Univariate analysis was conducted to explore factors predicting cannabis use in the survey cohort. Odds ratios were determined based on the survey question: *Have you ever used cannabis to manage your IBD symptoms*? Data from all respondents were included (n = 838) in the analysis of categorical survey items preceding this question (22 items; demographics, IBD characteristics, current satisfaction, QoL, and current clinical care).

A similar analysis of the data was also conducted to determine what factors predicted self-reported benefit of cannabis use in current/previous users. Odds ratios were determined based on the question: *Do you consider medicinal cannabis successful in managing your IBD symptoms*? Data from current and previous cannabis users were included (n = 212) in this analysis that involved categorical survey items (40 items).

### Definitions

#### Medicinal cannabis

This term is used as generally understood by laypeople in the community, namely, the use of cannabis to treat a specific disease or condition, as opposed to the recreational use of cannabis. The term does not imply that the use of cannabis has been authorized or prescribed by a medicinal practitioner, or that there is any evidence of cannabis being efficacious for a particular condition.

#### Therapeutic goods administration

The therapeutic goods administration (TGA) is Australia’s regulatory government body (akin to the FDA in the United States and EMA in EU) responsible for approving and regulating the use of all therapeutic goods (medicines and devices) in Australia. Under current legal MC access schemes in Australia, the TGA approves formal clinician requests for access to specific unregistered MC products for patients with specific refractory conditions.

#### Freedom of Information requests

Under the Freedom of Information Act 1982^32^ individuals may legally request access to government documents. We have routinely submitted Freedom of Information requests for access to current Australian MC prescribing data from the TGA to monitor the indications for which MC is being approved and numbers of approvals over time.

### Ethical Considerations

The survey was approved by the University of Sydney Human Research Ethics Committee (Ref: 2018/989) and carried out according to the National Statement on Ethical Conduct in Human Research (2007). All participants were required to acknowledge they had read the linked Participant Information Statement and to confirm their consent to the study before initiating the survey through REDCap.

## RESULTS

### Study Population

A total of 969 respondents consented and initiated a survey response. A total of 131 were excluded from the final analysis for the following reasons: (1) respondent did not confirm reading the Participant Information Statement and Consent Form (n = 55), (2) respondent was younger than 18 years of age (n = 3), (3) respondent did not have IBD (n = 19), or (4) respondent did not complete the minimum 35 required survey items (n = 54). The final data set comprised 838 respondents of which 67% (n = 556) completed all survey items [n = 403 nonusers (65% of nonuser cohort); n = 153 users (72% of user cohort)]. All included respondents (n = 838) completed a minimum of 35 items (including demographics, IBD severity, clinical care, current treatment satisfaction, cannabis use status, and QoL measures).

Respondents were asked if they currently use or had previously used cannabis to manage their IBD: 152 (18.1%) were current users, 60 (7.2%) were previous users of cannabis, and 626 (74.7%) reported being nonusers of cannabis for IBD management (Table 1).

**TABLE 1.** Demographics of Survey Population Grouped by Cannabis Use for IBD

			Cannabis Users			
		Nonusers	Current Users	Previous Users	All Users Combined	All Users vs Nonusers*
Age, mean (SD)		36.7 (11.6)	35.6 (10.9)	37.6 (10.8)	36.0 (11)	0.55
Sex, n (%)	Male	158 (23%)	60 (40%)	19 (68%)	79 (37%)	**0.001**
	Female	464 (74%)	91 (60%)	41 (32%)	132 (62%)	**0.001**
	Other	3 (0.5%)	0 (0%)	0 (0%)	0 (0%)	N/A
State/territory, n (%)	NSW	241 (39%)	47 (31%)	23 (38%)	70 (33%)	0.15
	QLD	120 (19%)	37 (25%)	14 (23%)	51 (24%)	0.13
	VIC	142 (23%)	38 (25%)	13 (22%)	51 (24%)	0.68
	NT	5 (0.8%)	0 (0%)	0 (0%)	0 (0%)	N/A
	WA	51 (8%)	11 (7%)	5 (8%)	16 (8%)	0.78
	SA	40 (7%)	9 (6%)	3 (5%)	12 (6%)	0.65
	TAS	10 (2%)	6 (4%)	1 (2%)	7 (3.3%)	0.13
	ACT	13 (2%)	2 (1%)	1 (2%)	3 (1.4%)	0.54
Ethnicity, n (%)	Caucasian/European	541 (87%)	137 (91%)	57 (98%)	194 (92%)	0.051
	Indigenous Australian or Torres Strait Islander	21 (3%)	3 (2%)	1 (2%)	4 (2%)	0.28
	Middle Eastern	19 (3%)	2 (1%)	0 (0%)	2 (0.9%)	0.09
	Jewish	15 (2%)	4 (3%)	0 (0%)	4 (2%)	0.67
	Chinese	4 (0.6%)	0 (0%)	0 (0%)	0 (0%)	N/A
	Indian	14 (2%)	0 (0%)	0 (0%)	0 (0%)	N/A
	Other	6 (1%)	5 (3%)	0 (0%)	5 (2.4%)	0.12
Highest level of education, n (%)	Primary	4 (0.6%)	2 (1%)	1 (2%)	3 (1%)	0.28
	Secondary	188 (30%)	45 (30%)	15 (25%)	60 (28%)	0.63
	Trade or vocational	192 (31%)	67 (44%)	19 (32%)	86 (41%)	**0.008**
	University degree	239 (38%)	37 (25%)	25 (42%)	62 (29%)	**0.019**
	Other	1 (0.2%)	0 (0%)	0 (0%)	0 (0%)	N/A
Employment, n (%)	Employed	484 (77%)	114 (75%)	46 (76.7%)	160 (76%)	0.58
Employment status, n (%)	Full time work	284 (59%)	73 (64%)	33 (72%)	106 (50%)	0.24
	Casual/part time work	171 (35%)	38 (33%)	7 (15%)	45 (21%)	0.080
	Student	19 (4%)	3 (3%)	4 (9%)	7 (3%)	0.85
	Other	9 (2%)	0 (0%)	2 (4%)	2 (1%)	0.65
Relationship status, n (%)	Partnered	452 (72%)	102 (67%)	44 (73%)	146 (69%)	0.35
Cigarette smoking, n (%)	Yes	57 (9%)	35 (23%)	8 (13%)	43 (20%)	**<0.0001**
	No	453 (72%)	78 (51%)	36 (60%)	114 (54%)	**<0.0001**
	Previously, not current	115 (18%)	39 (26%)	16 (27%)	55 (26%)	**0.018**

Bolded values represent statistically significant findings (P < 0.05).

Number of respondents varied per item; Nonusers = 483–626, Previous users = 46–60, Current users = 114–152. Percentage of respondents is reported for each item.

*Analysis of nonusers versus all users (previous and current users combined). Continuous variables were compared with Student’s *t*-test and discrete variables were compared with Pearson’s χ ^2^ test with Bonferroni correction.

N/A, χ ^2^ analysis not able to be computed for comparisons as value less than 1.

Demographic characteristics of the survey population are reported in Table 1 according to the cannabis use category (current, previous, or nonuser). Responses were received from all Australian states and territories. The majority of respondents were employed (76.9%), many on a fulltime basis (46.5%), partnered (71.6%), and most were not tobacco smokers (68.7%). Participants reported hearing about the survey primarily through Facebook (77.3%) and Twitter (7.3%) (Supplementary Table S1*provides a detailed breakdown of recruitment sources for the entire survey cohort*).

### Disease Severity and QoL

The clinical characteristics of the survey population (n = 838) are reported in Table 2. CD was the most frequently reported type of IBD (64.7%), followed by UC (31.0%) and IBDU (4.3%) (see Supplementary Table S2*for clinical characteristics of IBDU respondents*).

**TABLE 2.** Clinical Characteristics of Respondents by IBD Type

		Crohn’s Disease			Ulcerative Colitis			All IBD^b^		
		Nonusers	All Users^a^	P*	Nonusers	All Users^a^	P*	Nonusers	All Users^a^	P*
BMI, n (%)	Underweight (<18.5)	21 (5.3%)	5 (3.4%)	0.34	4 (1.9%)	0 (0%)	^N/A^	25 (4.0%)	6 (2.8%)	0.44
	Healthy (18.5 to <25)	147 (37.3%)	56 (37.8%)	0.91	83 (40.3%)	19 (40.4%)	0.94	234 (37.4%)	83 (39.2%)	0.65
	Overweight (25 to <30)	124 (31.5%)	47 (31.8%)	0.95	68 (33%)	20 (42.6%)	0.25	201 (32.1%)	72 (34%)	0.62
	Obese (≥30)	95 (24.1%)	37 (25%)	0.83	51 (24.8%)	8 (17%)	0.24	154 (24.6%)	46 (21.7%)	0.39
Disease years, mean (SD)		11.25 (10.35)	12.68 (9.25)	0.14	7.94 (7.43)	7.73 (4.95)	0.85	10.05 (9.56)	11.51 (8.91)	0.051
Under the care of a GP for IBD? n (% yes)		326 (84.2%)	127 (87.6%)	0.41	163 (79.9%)	37 (78.7%)	0.84	505 (82.7%)	177 (85.9%)	0.27
GP visits per year, mean (SD)		6.95 (6.19)	8.27 (7.14)	0.052	6.88 (6.34)	6.7 (6.90)	0.88	7.02 (6.35)	8.01 (7.22)	0.086
Under the care of a specialist for IBD? n (% yes)		369 (93.9%)	130 (87.8%)	**0.019**	195 (93.3%)	40 (83.3%)	**0.041**	577 (92.8%)	177 (83.1%)	**<0.0001**
Specialist visits per year, mean (SD)		4.92 (3.023)	4.23 (3.06)	**0.026**	4.43 (3.53)	3.95 (3.34)	0.43	4.16 (3.22)	4.11 (3.10)	0.86
Current use of pharma drugs for IBD? n (% yes)		340 (87.4%)	119 (81.0%)	0.072	189 (91.3%)	37 (78.7%)	**0.020**	539 (87.5%)	161 (77.0%)	**0.0003**
MARS, mean (SD)		6.68 (2.20)	6.22 (2.36)	**0.037**	6.54 (2.07)	6.33 (2.28)	0.55	6.66 (2.17)	6.21 (2.34)	**0.014**
Hospitalized for your IBD? n (% yes)		327 (83%)	132 (89.2%)	0.082	119 (57%)	34 (69%)	0.11	458 (73.4%)	166 (81%)	**0.016**
No. hospitalizations since diagnosis, mean (SD)		5.49 (3.66)	6.57 (3.72)	**0.005**	3.54 (2.51)	3.85 (2.56)	0.53	4.97 (3.49)	5.95 (3.64)	**0.002**
Undergone surgery for IBD? n (% yes)		205 (52.0%)	76 (51.4%)	0.92	10 (5%)	3 (6%)	0.72	221 (35.3%)	81 (38.2%)	0.46
SIBDQ, mean (SD)		37.02 (11.45)	36.26 (11.86)	0.72	37.84 (12.54)	40.87 (12.16)	0.13	37.18 (11.81)	36.96 (11.83)	0.82
EQ-5D-5L, utility score, mean (SD)		0.578 (0.203)	0.529 (0.246)	**0.019**	0.593 (0.224)	0.614 (0.206)	0.55	0.579 (0.214)	0.564 (0.236)	0.059
EQ-5D-5L, health today (of 100), mean (SD)		59.34 (20.60)	55.78 (20.92)	0.075	61.65 (20.76)	68.8 (17.14)	**0.026**	59.92 (20.87)	59.13 (20.57)	0.63
WPAI, overall work impairment, % (SD)		28.07 (31.05)	24.81 (29.96)	0.27	24.79 (29.55)	26.61 (33.50)	0.71	27.11 (30.89)	25.94 (30.8)	0.63
WPAI, activity impairment, % (SD)		41.78 (29.26)	44.66 (28.91)	0.31	39.24 (29.10)	33.88 (32.33)	0.065	41.13 (29.27)	42.26 (29.52)	0.63

Bolded values represent statistically significant comparisons (P > 0.05).

Number of respondents: CD nonusers = 394, CD all users = 148, UC nonusers = 211, UC all users = 49, all IBD nonusers = 626, all IBD users = 212.

^a^All users column combines previous and current MC users.

^b^All IBD includes all survey respondents (UC, CD, and IBDU).

*P-value represents analysis of nonusers versus all users for each IBD type (UC, CD, all IBD). Continuous variables were compared with Student’s *t*-test and discrete variables were compared with χ ^2^ analysis.

N/A, χ ^2^ analysis not able to be computed for comparisons as value less than 1.

SIBDQ, Short Inflammatory Bowel Disease Questionnaire; MARS, Medication Adherence Rating Scale; WPAI, Work Productivity and Activity Impairment Questionnaire; EQ-5D-5L, EuroQoL Five dimension Five level Questionnaire.

Clinical disease severity as measured by modified disease activity indices (Supplementary Table S3) was not significantly different between users and nonusers of cannabis in any of the IBD categories (Table 2). However, cannabis users (current and previous combined) were more likely to have been hospitalized for their IBD and those who had been hospitalized reported a greater number of hospitalizations since IBD diagnosis than nonusers (χ ^2^ (1, 5.76), *P* < 0.05 and *t*(617) = 3.1, *P* < 0.01, respectively, Table 2).

Cannabis users had a lower MARS (medication adherence) score compared to nonusers (6.21/10 vs 6.66/10, respectively; *t*(777) = 2.47, *P* < 0.05). Overall, users had a lower prevalence of use of pharmaceutical drugs (77.0% in users vs 87.5% in nonusers; χ ^2^ (1, 13.3), *P* < 0.001) and less IBD specialist engagement than nonusers (83.1% users vs 92.8% nonusers; χ ^2^ (1, 16.93), *P* < 0.0001, Table 2).

There were no differences in reported QoL between cannabis users and nonusers (EQ-5D-5L and SIBDQ scores, Table 2). Nor did they report differing degrees of activity impairment (WPAI, Table 2).

When only CD respondents were considered, cannabis users reported more hospitalizations (*t*(447) = 2.84, *P* < 0.01), less medication adherence (MARS score; *t*(507) = 2.10, *P* < 0.05), and less engagement with and fewer visits to a gastroenterological specialist (engagement, χ ^2^ (1, 5.5), *P* < 0.05; frequency of visits, *t*(495) = 2.23, *P* < 0.05, Table 2). In addition, cannabis users with CD reported a poorer overall QoL (EQ-5D-5L utility score, *t*(540) = 2.36, *P* < 0.05, Table 2).

When only UC respondents were considered, cannabis users were less likely to be under the care of a specialist (Fisher’s exact *P* < 0.05) and less likely to be taking prescription drugs for their IBD (Fisher’s exact *P* < 0.05). However, cannabis users with UC reported significantly better QoL than their nonusing counterparts on one snapshot measure (EQ-5D-5L health today *t*(258) = 2.24, *P* < 0.05, Table 2).

### Current IBD Symptom Management

Respondents endorsed one of 5 responses describing their current satisfaction (“very dissatisfied” to “very satisfied”) with IBD symptom management (Supplementary Figure S1) and their currently prescribed pharmaceuticals (Table 3). These responses were strategically elicited before any questions about cannabis use to minimize “expectational” responses in users, with later items in the survey specifically addressing changes in symptoms and satisfaction specific to cannabis use.

**TABLE 3.** Current IBD Pharmaceutical Satisfaction as Reported by Cannabis Users and Nonusers

IBD Drug Class	Current Satisfaction Level^a^	Nonusers	All Users^b^	P*
Calcineurin inhibitors	Satisfied	3 (27%)	1 (33%)	NA
	Neutral	2 (18%)	1 (33%)	
	Dissatisfied	**6 (55%)**	1 (33%)	
Biologic agents	**Satisfied**	**163 (67%)**	**48 (55%)**	0.12
	Neutral	39 (16%)	18 (20%)	
	Dissatisfied	42 (17%)	22 (25%)	
Antidepressants/anxiolytics	Satisfied	**68 (55%)**	19 (34%)	**0.002**
	Neutral	32 (26%)	13 (23%)	
	Dissatisfied	23 (19%)	**24 (43%)**	
Benzodiazepines	**Satisfied**	**19 (40%)**	**15 (45%)**	0.51
	Neutral	17 (36%)	8 (24%)	
	Dissatisfied	11 (23%)	10 (30%)	
Antibiotics	Satisfied	18 (32%)	7 (25%)	**0.048**
	Neutral	19 (34%)	4 (14%)	
	Dissatisfied	19 (34%)	**17 (61%)**	
Aminosalicylates	**Satisfied**	**134 (51%)**	**22 (41%)**	0.31
	Neutral	53 (20%)	15 (28%)	
	Dissatisfied	74 (28%)	17 (31%)	
Immunomodulators	**Satisfied**	**144 (53%)**	**38 (46%)**	0.56
	Neutral	55 (20%)	19 (23%)	
	Dissatisfied	75 (27%)	26 (31%)	
Corticosteroids	Satisfied	65 (35%)	**29 (49%)**	0.12
	Neutral	34 (18%)	7 (12%)	
	Dissatisfied	**89 (47%)**	23 (39%)	
Analgesics	Satisfied	**104 (42%)**	31 (32%)	0.15
	Neutral	82 (33%)	33 (34%)	
	Dissatisfied	62 (25%)	33 (34%)	

Bolded values indicate the majority satisfaction level for each user group within in each drug class.

Number of respondents for each group varies based on the number having experience with that pharmaceutical class. Number of responses (n = users, n = nonusers): calcineurin inhibitors (3, 11), biologic agents (88, 244), antidepressants/anxiolytics (56, 123), benzodiazepines (33, 47), antibiotics (28, 56), aminosalicylates (54, 261), immunomodulators (83, 274), corticosteroids (59, 188), and analgesics (97, 248).

^a^Satisfied = sum of “very satisfied” + “somewhat satisfied.” Dissatisfied = sum of “very dissatisfied” + “somewhat dissatisfied” responses.

^b^All users = combined past and current cannabis users.

*From χ ^2^ contingency test.

NA, not applicable—unable to calculate *P*-value with n = 1 in all users group.

Overall, cannabis users (current and previous combined) and nonusers reported similar satisfaction with their current symptom management (χ ^2^ (4, 2.587), *P* > 0.5, Supplementary Figure S1) and their current pharmaceutical regimen (Table 3) with the exception of antidepressant/anxiolytic and antibiotic satisfaction. Cannabis users were less satisfied with both drug classes for their IBD management than nonusers (χ ^2^ (2, 12.28), *P* < 0.01, and χ ^2^ (2, 6.08), *P* < 0.05, respectively). However, the reported rates of use of these 2 classes of drug were higher in cannabis users than nonusers (34.8% use in cannabis users vs 22.9% use in nonusers of antidepressants/anxiolytics, χ ^2^ (1, 9.16), *P* < 0.005; 17.4% vs 10.4% use of antibiotics, χ ^2^ (1, 5.67), *P* < 0.05, respectively).

### Characteristics of Cannabis Use

**FIGURE 1.**
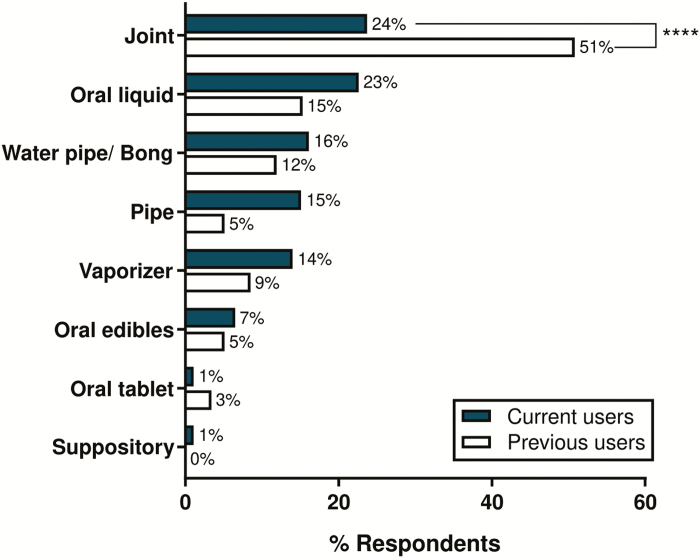
The most common method of cannabis use reported for previous and current cannabis users to manage their IBD symptoms (n = 93 current users; n = 59 previous users). Previous users were significantly more likely to use joints compared to current users (χ ^2^ (1, 29.94), *P* < 0.0001). Percentage of total respondents shown for this item.

The most common routes of cannabis administration were via smoking a joint (34.2%), oral liquid (19.7%), or smoking via a water pipe/bong (14.5%) with the least common route being a suppository (0.7%). The use of joints was greater in previous users compared to current users (χ ^2^ (1, 29.94), *P* < 0.0001, Figure 1).

Current users accessed their cannabis through a recreational dealer (44.6%), friends and family (26.1%), growing their own (9.8%), illicit suppliers of MC products (8.7%), online suppliers (5.4%), cannabis access clinics (2.2%), overseas suppliers (1.1%), prescription via a medical practitioner (1.1%), and other (1.1%). Only 3 respondents were using the legal pathways provided by the Australian government.

Current users medicated with cannabis near-daily (current users mean 30.0 ± 24.7 times per month; previous users 22.7 ± 23.5 times per month). Half of the current users also used cannabis for recreational purposes (recreational and medicinal use 51%, n = 47, medicinal use only 49%, n = 45). Recreational use tended to be less frequent than medicinal use (mean 14.33 ± 23.3 times per month). Of the co-recreational current users, only 19% (9/46) reported using cannabis less than 4 times per month for recreational purposes.

The decision to use cannabis was driven by discovering benefits on one’s own (57% of all users, n = 87) and through friends or family (20%, n = 31). Other reasons included discussions with conventional healthcare providers (5%, n = 7), alternative healthcare practitioners (2%, n = 3), MC advocacy groups (1.3%, n = 2), or no reason provided (14%, n = 22).

Perceived knowledge of the cannabinoid content of the preparations being used was inconsistent which is unsurprising, given the use of largely unregulated, illicit products; 44% of current users reported uncertainty regarding the content or variable content from batch to batch. Those endorsing knowledge of content reported THC-dominant products (20%), CBD-dominant (18%), or an equal ratio of THC:CBD (18%). Perceived content varied across IBD types (χ ^2^ (6, 17.07), *P* < 0.01) with UC respondents most likely to report use of THC-predominant products (55%), CD respondents reporting the highest rate of “unknown/variable content” products (44%), and IBDU patients most likely to report using a CBD-predominant product (40%).

### Symptom Relief With Cannabis Use

An overwhelming majority of all users (current and previous combined) reported their IBD had improved since using cannabis (UC 97.1% improved; CD 82.1%; IBDU 90.1%). A small proportion reported no change to their IBD due to cannabis (UC 2.9% no change; CD 11.3%; IBDU 9.1%). A few CD respondents (mostly previous users; 6.6%) reported their IBD as worse compared to before cannabis use.

**FIGURE 2.**
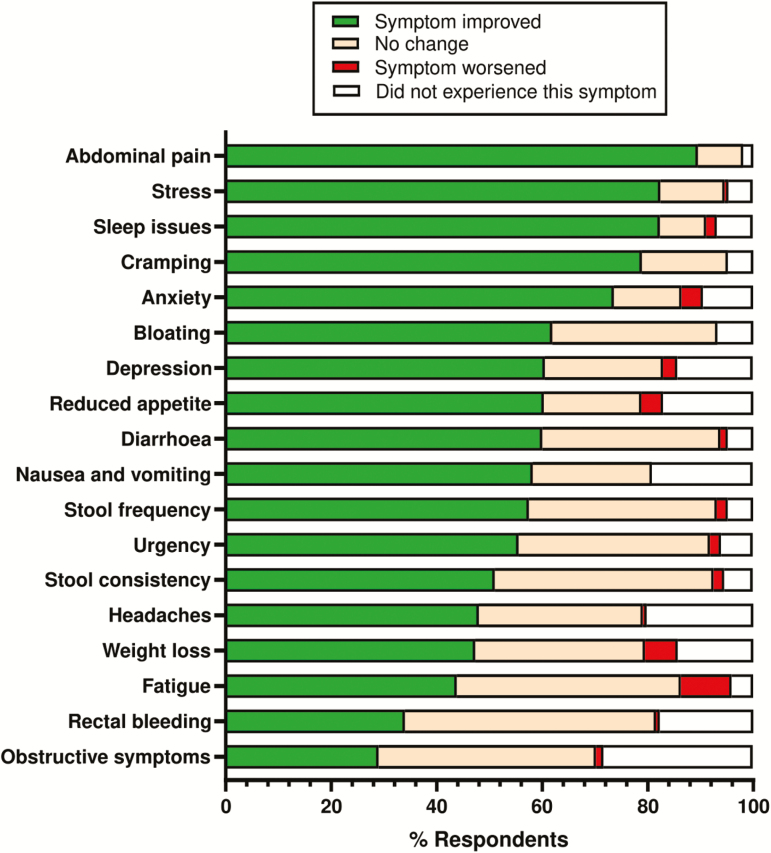
Self-reported IBD symptom change with medicinal cannabis use in current and previous users listed by greatest positive benefit to least. Green = positive change in symptom; orange = no change; red = negative change; gray = did not report having this symptom; n = 152 current users; n = 60 previous users. Percentage of total respondents shown.

Respondents reported greatest improvements in abdominal pain, stress, sleep issues, cramping, and anxiety with cannabis use, with the least improvement in obstructive symptoms, rectal bleeding, and fatigue (Figure 2). Symptomatic relief varied with IBD types. In UC, cannabis best improved sleep issues (100%), abdominal pain (97.1%), stress (94.1%), and anxiety (88.2%) (Supplementary Figure S2A), while CD users reported improvement in abdominal pain (96.2%), stress (92.5%), cramping (90.6%), and anxiety (88.7%) (Supplementary Figure S2B). Symptoms most frequently improved in IBDU were fatigue (100%), anxiety (100%), rectal bleeding (100%), and diarrhea (100%) (Supplementary Figure S2C). Within the top 5 improved symptoms, anxiety was common across all IBD types.

**FIGURE 3.**
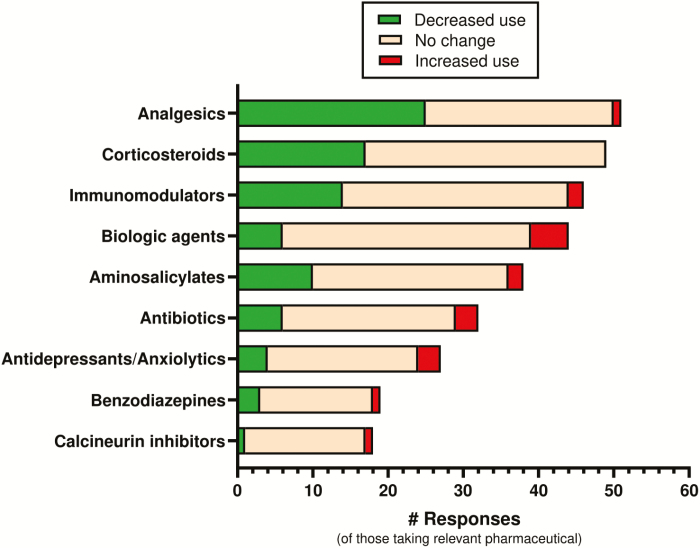
Current medicinal cannabis user’s self-reported change in pharmaceutical drug classes while using cannabis for their IBD. Data listed by greatest drug reductions to least. Green = reduced use of drug class; orange = no change in use; red = increased use of drug class. Number of respondents for each drug class varies based on the number of current cannabis users taking that class of drugs concomitantly.

Of those current cannabis users medicating with analgesics (59% of cohort), approximately half (49%, 25/51) reported reduced analgesic use as a result of their current cannabis use (Figure 3) with 21.6% (11/51) of patients reporting a “marked reduction” (>50% reduction). A significant proportion of respondents using corticosteroids (56% of cohort), immunomodulators (52% of cohort), and aminosalicylates (43% of cohort) reported reduced intake of that pharmaceutical class as a result of cannabis use (34.7%, 30.4%, and 26.3% reductions, respectively). There was little overall reported change in the use of antidepressants, anxiolytics, and biologic agents with cannabis use, with the majority (74.1%–78.9%) reporting no change (Figure 3).

When asked about the perceived success of MC for IBD management overall in current and past cannabis users, highest success rates were reported in respondents with UC (100%) followed by CD (90.6%) and IBDU (90.9%).

### Factors Predicting Cannabis Use

Table 4 shows the results of univariate analysis of factors determining a positive response to the question: *Have you ever used cannabis to manage your IBD symptoms*? Analysis showed that current and previous cannabis users were more likely to be male, with a trade/vocational education and also be present/past tobacco smokers. A diagnosis of UC predicted lower likelihood of cannabis use relative to CD and IBDU. Hospitalization from complications relating to IBD was a predictor of use, as was lifetime hospitalizations of greater than 10 times for IBD. Lack of any current pharmaceutical drug regimen for IBD management was a strong predictor of use, as was a lack of engagement with a gastroenterological specialist. Absence of alternative/complementary therapy use negatively predicted cannabis use. Comparison of current users who are medicinal only versus co-recreational/medicinal users did not yield any significant predictors.

**TABLE 4.** Predictors of “Cannabis Use” and Reported “Cannabis Benefit” From Survey Cohort Characteristics (Univariate Analysis)

Predictor		OR	95% CI	P*	Predictor Type
Cannabis Use					
Sex	Male	1.77	1.27–2.47	**0.0007**	+
	Female	0.58	0.42–0.81	**0.001**	−
Ethnicity	Caucasian/European descent	1.89	1.06–3.36	**0.030**	+
Highest education level	Trade/vocational	1.55	1.12–2.14	**0.008**	+
	University degree	0.67	0.48–0.94	**0.020**	−
Tobacco use	Past smoker	1.56	1.08–2.25	**0.018**	+
	Present smoker	2.49	1.62–3.83	**<0.0001**	+
	Non smoker	0.44	0.32–0.62	**<0.0001**	−
IBD diagnosis	Ulcerative colitis	0.59	0.41–0.85	**0.004**	−
	Crohn’s disease	1.36	0.97–1.90	0.071	ns
	IBD unspecified	2.19	1.1–4.33	**0.024**	+
Hospitalizations for IBD	Hospitalized	1.61	1.09–2.37	0.018	+
	Never hospitalized	0.62	0.42–0.92	0.018	−
	1–3 times in lifetime	0.54	0.38–0.78	**0.001**	−
	>10 times in lifetime	1.72	1.14–2.59	**0.010**	+
Clinical care (IBD specific)	No pharmaceutical regimen	2.09	1.4–3.12	**0.0003**	+
	Current pharmaceutical regimen	0.48	0.32–0.72	0.0003	−
	No specialist care	2.46	1.53–3.97	**0.0002**	+
	Under specialist care	0.41	0.25–0.65	0.0002	−
Complementary/alternative therapies	No use of any alternative therapies	0.11	0.05–0.22	**<0.0001**	−
Cannabis Benefit					
Highest education level	University degree	0.16	0.04–0.66	**0.011**	−
Cannabis use status	Previous	0.21	0.05–0.83	**0.026**	−
	Current	4.75	1.2–18.7	0.026	+
Complementary/alternative therapies	Chiropractic/osteopathy/massage	0.25	0.07–0.88	**0.032**	−
	Restriction/exclusion dieting	0.25	0.06–0.97	**0.044**	−
Type of cannabis used	CBD-only product	0.01	0.008–0.35	**0.008**	−

Only predictors that were significant (*P* < 0.05) were reported from the dataset of survey items.

*P-values calculated using a χ ^2^ test for independence.

OR, odds ratio; CI, confidence interval; ns, not significant.

### Factors Predicting Benefits of Cannabis Use

Table 4 shows the results of univariate analysis of factors predicting a positive response in current and previous cannabis users (n = 212) to the question: *Do you consider medicinal cannabis successful in managing your IBD symptoms*?

Unsurprisingly, current cannabis use was a positive predictor of perceived benefit, while being a previous user was a negative predictor. Education level predicted use and also perceived benefit, with those having completed a university degree being less likely to report benefits of cannabis.

However, the use of some other alternative therapies was a negative predictor of cannabis benefit in IBD (ie, chiropractic/osteopathy/massage and restriction/exclusion dieting), as was perceived cannabinoid content, with those thought to be using CBD-only products less likely to report benefit.

### Side Effects in Cannabis Users

Table 5 shows the side effects experienced by current and previous users. There were very few severe or intolerable side effects reported although mild and tolerable side effects were common. The most common reported side effects were increased appetite (74%), dry mouth (64%), drowsiness or sedation (59%), memory impairment (34%), and lack of energy or fatigue (34%). Previous users were significantly more likely than current users to report anxiety and delusions, potentially contributing to their cessation of use (Table 5, Fisher’s exact test; *P* < 0.05).

**TABLE 5.** Side Effects Experienced by Both Current and Previous Cannabis Users

	Mild and Tolerable Side Effects		Severe or Intolerable Side Effects		
	Current Users	Previous Users	Current Users	Previous Users	P*
Increased appetite	62 (68%)	40 (70%)	7 (8%)	0 (0%)	0.54
Dry mouth	59 (64%)	31 (54%)	4 (4%)	1 (2%)	0.13
Drowsiness or sedation	50 (55%)	33 (58%)	3 (3%)	1 (2%)	0.87
Memory impairment	30 (33%)	17 (30%)	1 (1%)	2 (4%)	>0.99
Lack of energy or fatigue	26 (29%)	20 (35%)	5 (5%)	0 (0%)	0.90
Dehydration	19 (21%)	10 (18%)	2 (2%)	0 (0%)	0.54
Confusion	18 (20%)	9 (16%)	1 (1%)	0 (0%)	0.52
Dizziness	16 (17%)	9 (16%)	0 (0%)	0 (0%)	>0.99
Bad taste in mouth	15 (16%)	12 (21%)	2 (2%)	3 (5%)	0.30
Diarrhea	14 (15%)	8 (14%)	2 (2%)	0 (0%)	0.83
Anxiety	14 (15%)	17 (30%)	1 (1%)	1 (2%)	**0.042**
Eye irritation	13 (14%)	6 (11%)	0 (0%)	1 (2%)	0.81
Respiratory complaints	9 (10%)	3 (5%)	0 (0%)	3 (5%)	>0.99
Racing heart/palpitations	9 (10%)	5 (9%)	0 (0%)	2 (4%)	0.79
Paranoia	9 (10%)	9 (16%)	0 (0%)	2 (4%)	0.14
Gastrointestinal irritation	9 (10%)	5 (9%)	0 (0%)	0 (0%)	>0.99
Depressed mood	9 (10%)	6 (11%)	1 (1%)	0 (0%)	>0.99
Nausea/vomiting	8 (9%)	3 (5%)	2 (2%)	0 (0%)	0.37
Sleep disturbance	7 (8%)	4 (7%)	2 (2%)	1 (2%)	>0.99
Constipation	6 (7%)	3 (5%)	1 (1%)	0 (0%)	0.74
Sweating	5 (5%)	3 (5%)	1 (1%)	1 (2%)	>0.99
Headaches	5 (5%)	5 (9%)	2 (2%)	0 (0%)	0.77
Allergies	4 (4%)	1 (2%)	0 (0%)	0 (0%)	0.65
Shaking/tremor/poor movement control	3 (3%)	3 (5%)	0 (0%)	1 (2%)	0.43
Decreased appetite	3 (3%)	2 (4%)	1 (1%)	0 (0%)	>0.99
Panic attack	2 (2%)	1 (2%)	0 (0%)	1 (2%)	0.64
Nasopharyngeal complaints	2 (2%)	3 (5%)	1 (1%)	0 (0%)	0.68
Cannabis hyperemesis	2 (2%)	2 (4%)	1 (1%)	0 (0%)	>0.99
Hallucinations	1 (1%)	4 (7%)	0 (0%)	0 (0%)	0.072
Delusions	0 (0%)	6 (11%)	1 (1%)	0 (0%)	**0.014**

Number of respondents; current users = 89–92; previous users = 56–57.

*P value represents contingency test between current and previous users of all side effects experienced (severe and mild combined) using a χ ^2^ analysis or Fisher’s exact test where n < 20.

### Factors Determining Cessation of Cannabis Use and Nonuse

Previous users cited difficulty accessing cannabis as the main reason for discontinuing use (29.8%) and endorsed a higher rate of difficulties in obtaining cannabis than current users (47% of current users; 83% of previous users, χ ^2^ (1, 18.02), *P* < 0.0001). Other factors driving cessation of use included concerns around illegality (19.3%), concerns around roadside drug testing (17.5%), and interference with job and/or social life (7.0%). An oversight in the survey design meant that “side effects” were not provided as an explicit option in response to the item interrogating why cannabis use had ceased. Nonetheless, as noted above, significantly more side effects were experienced by previous users than current users, suggesting this may be an important factor (Table 5). Perhaps surprisingly, all previous users (100%) expressed interest in using cannabis in the future to treat IBD symptoms despite their discontinuation of cannabis use.

Nonusers reported a range of concerns that maintained their abstinence from cannabis, including concerns about illegality (48%), difficulties buying or accessing cannabis (37%), concerns about roadside drug testing (35%), and concerns about interference with the job and/or social life (32%). Of the 93 (20%) participants who responded in the “Other” category, reasons included lack of awareness of MC being an option (45%), concern about side effects (16%), insufficient evidence to support use in IBD (15%), and disease currently well-controlled (15%).

### Attitudes Toward Legality and Future Access

The majority of current (73%) and previous cannabis users (63%) supported the full legalization of cannabis for all purposes, while only a minority of nonusers supported this (31% nonuser support). A majority of nonusers (62%) preferred cannabis be legal only for medicinal purposes (compared to 25% of current users and 35% of previous users). Several respondents reported being unsure (5% nonusers; 1% current users) or having no opinion (2% nonusers only) on legal status. Only 2 respondents (both users, 0.4%) asserted it should be illegal for all purposes.

Willingness to trial MC to treat their IBD was extremely high among nonusers (94%) and previous users (100%), suggesting likely selection bias within the respondent population. This level of willingness may not therefore be reflective of the broader Australian IBD cohort.

The preferred routes of cannabis consumption for interested nonusers were oral tablet/capsules (66%), oral liquids (10%), and oral edibles (9%). A high proportion of respondents expressed interest in participating in future clinical trials of MC products (current and previous combined, 82%) and nonusers (75%).

## DISCUSSION

To our knowledge, this is the largest survey to date on cannabis use in patients with IBD.^20–24^ It provides insights into the perceived benefits and side effects of cannabis use, the interaction with conventional medical treatment, and the factors that may drive continuing use, discontinuation, and nonuse of cannabis, all from a patient perspective.

Overall, cannabis users seemed less likely to be engaged in clinical treatment for their IBD, with less likelihood of being under specialist care, lower likelihood of pharmaceutical drug use, and poorer medication adherence. Lack of engagement with conventional care in MC users might be explained in several ways. One is that a proportion of patients with refractory disease (who may have trialed many existing evidence-based options) have, in some ways, given up on conventional medications and are exploring alternatives such as MC. It may also be the case that patients who have never been well engaged in clinical care are utilizing MC as a substitute for prescription medications.

We did not collect data to indicate that refractory patients had exhausted all approved options before trialing MC. Despite this, the fact that 76% of MC users (n = 161/212) still take prescribed IBD medications concomitantly (confirming they are under some form of engaged care) does suggest some refractory state and inadequate control of their disease. In other studies MC use was predicted by greater severity and chronicity of disease, presumably motivating a desperate search for alternative therapies.^33^

Furthermore, being frequently hospitalized (>10 times) for IBD positively predicted MC use, while fewer hospitalizations (0–3 times in a lifetime) negatively predicted use. This is consistent with at least one previous report.^21^ However, there is ambiguity in this result. Increased numbers of hospitalizations for IBD may infer increased disease severity although it is notable that current MC users did not differ in current disease severity compared to nonusers based on the survey responses.

The alternative possibility is that less engagement with clinical care and poor medication adherence drives increased hospitalizations in MC users. Epidemiological studies report that recreational cannabis use in IBD patient cohorts was associated with reduced length of hospital stays and less hospital charges as a result.^34^ However, there are scarce data on the number of hospitalizations being affected by purposive MC use, and interpretations relating to this are speculative. Reports assessing the effect of cannabis legalization (as a proxy for increased community cannabis use) on hospitalizations suggests neutral effects.^35^

### Symptom Control Versus Disease Modification With Cannabis

Cannabis users self-reported significant symptomatic relief with improvements in abdominal cramping and pain. This agrees with previous reports in CD patients using cannabis^21^ and has a plausible mechanistic basis.^36^ Many of the symptoms reported as improved were not directly related to IBD pathophysiology and reflected psychological comorbidities such as stress, sleep issues, and anxiety (Figure 2). Indirect benefits of MC on sleep are widely described in other conditions such as neuropathic pain and Parkinson’s disease and anxiety.^37^ Improvements in sleep can affect how patients perceive pain, anxiety, and stress (and vice versa)^38^ and may also have positive effects on inflammation in IBD.^39^ Sleep was the primary symptom improved in this UC cohort in addition to abdominal pain, and this was consistent across the 3 IBD types.

Improvements were less commonly reported in symptoms associated with disease progression such as rectal bleeding, obstructive symptoms, and stool frequency/consistency/urgency (Figure 2). This suggests that MC may potentially be modifying comorbid symptoms (ie, anxiety, pain, and sleep) or at least the patient’s perception of these symptoms, more than altering disease progression, consistent with the mixed results of clinical trials conducted to date. It is also worth considering whether reduced use of pharmaceuticals (with significant side effect burdens) (Figure 3) may also explain symptomatic improvements in cannabis users. Reported reductions in the use of key IBD drug classes (ie, immunomodulators, aminosalicylates, and corticosteroids) in cannabis users agree with the results of a recent observational report of IBD patients using legally prescribed MC,^17^ supporting this possibility.

High rates of side effects were reported in MC users (Table 5), most notably drowsiness/sedation and memory impairment. These may be a significant impediment to long-term use of MC in IBD and other conditions, as is widely acknowledged.^5, 40^ The majority of side effects reported are characteristic of THC intoxication,^7^ as opposed to CBD. Obviously the doses of THC being used by respondents in the current survey are unknown, but given the sourcing of products from recreational dealers, it is likely to be considerable (given the high % THC content in street cannabis^41–43^).

Despite the self-reported symptomatic improvement, QoL showed a complex relationship with cannabis use within our survey cohort (Table 2, Supplementary Table S2). Improved QoL with cannabis use was observed in the UC cohort on one nonspecific measure (EQ-5D health today item), while cannabis users with CD actually reported lower overall QoL than nonusers (as measured by overall EQ-5D utility score that encompasses 5 dimensions of QoL). However, in neither case were these small changes (in EQ-5D scores) supported by a difference in the health-specific QoL measure, SIBDQ, suggesting these differences may not be clinically meaningful. Furthermore, interpretation of these findings is challenging: by sampling at only a single time point it is difficult to infer if QoL differed at baseline user and nonuser groups or whether it has been affected by MC use. We expect that the former is the case given that only 14% of users reported no change or worsening of their condition overall from cannabis use (which we expect to reflect an improved QoL).

It is notable that significant improvements in QoL have been reported in 2 clinical trials of cannabis for IBD^44^ and in advanced cancer patients,^45^ including anecdotal reports (from these trials) of improved appetite and sleep—which supports our findings on symptom improvement (Figure 2).

Finally, it should be noted that the magnitude of self-reported symptom improvement in the present survey was surprisingly high. Symptom improvements of the magnitude reported in this study are rarely seen in any therapeutic intervention and do not easily align with the existing randomized controlled trial results of cannabinoids in IBD cohorts. This emphasizes the need for independent clinical validation of the symptom improvements reported by respondents and raises the possibility of some selection bias within the study cohort.

### Interaction Between Medicinal and Recreational Cannabis Use

Cannabis users in our study cohort tended to be male, Caucasian, non-university educated, and tobacco smokers; all factors known to predict recreational cannabis use.^46, 47^ Indeed, 51% of the current user sample consumed recreationally on a regular basis, which likely explains how they “discovered (medical) benefits on my own.” However, the recreational use was less frequent than medicinal use, with the latter occurring daily in the majority of current users. The most common source of cannabis was through a recreational dealer, further blurring the delineation between medicinal and recreational use.

Those exclusively endorsing medicinal use favored CBD-dominant oral liquid products compared to the more prevalent use of recreational modalities (joints/bongs) in combined recreational/medicinal users. Notably, nonusers strongly endorsed pill/capsule use in the anticipated future use of MC products with only a small minority prefering traditional recreational methods (ie, joint). This again highlights differences between purposive current users, interested nonusers, and those using cannabis medicinally as a result of preexisting recreational use.

Targeted surveys of patients receiving MC through the legal government scheme would be the ideal sample to evaluate perceived benefit, as the content and dose of the product are regulated and verifiable, and there is clinical supervision and evaluation of their MC use. Notably, only 3 respondents reported accessing cannabis products from official sources, consistent with contemporary criticism of access pathways in Australia.^48^

### Limitations

There are several limitations to our survey to acknowledge. Anonymous, open-access, online surveys have inherent limitations related to sampling bias, reliability of responses, and an inability to verify the clinical diagnosis and disease severity. Assertions regarding the efficacy of cannabis cannot be independently verified, and neither can the content of products perceived as effective/ineffective (especially given that >98% of products used were from unregulated sources). While our recruitment strategy focused on IBD patients rather than cannabis users, and recruited only a relatively small proportion of respondents who use MC (25%), it remains possible that the nonusers recruited were in some way more sympathetic toward MC than the broader population of IBD patients.

Finally, future surveys might usefully ask whether patients: *Are comfortable in discussing cannabis with your doctor/specialist*? Other surveys within Australia^25^ suggest that the proportion of patients willing to discuss cannabis use with their doctor is low (36%).

## CONCLUSION

This survey has described the current landscape of MC use in an Australian IBD population. From the knowledge gained it is likely there are 2 populations of IBD patients (with overlapping use patterns and clinical features) using MC. The spectrum of patients encompasses existing cannabis users who have limited engagement with traditional clinical care (ie, pharmaceuticals/clinicians) and who may be influenced by expectational bias, ranging to purposive medicinal users with potentially refractory cases of IBD desperately seeking and trialing alternatives to manage their symptoms. We recognize a large proportion of IBD cannabis users likely fall somewhere between these 2 ends of the spectrum making analysis of cannabis use/benefit in uncontrolled clinical cohorts challenging. The fact that only 3/212 respondents were using legal, regulated MC products further challenges this analysis.

In the absence of effective disease-modifying drugs, control of negative comorbid symptoms (ie, pain, anxiety, and sleep issues) and reduction of pharmaceutical burden are beneficial for a patient’s QoL and ability to cope with their chronic disease status; this is the gap that cannabis seems to be filling for some Australians with IBD based on their self-report. While preclinical investigations continue into novel cannabinoids and their potential to treat the underlying IBD pathophysiology, it is important that clinical investigations into the ability of adjunct MC to reduce symptom burden in IBD populations continue to validate or refute patient claims. This is especially prudent as there are now legal access pathways for regulated quality MC products in Australia.

## Supplementary Material

otaa015_suppl_Supplementary_Tables

otaa015_suppl_Supplementary_Figure_S1

otaa015_suppl_Supplementary_Figure_S2

otaa015_suppl_Supplementary_Material_REDCAP

otaa015_suppl_Supplementary_Material_Advertising

## Abbreviations (non-standard)

MC: medicinal cannabis
IBDU: inflammatory bowel disease unspecified
CBD: cannabidiol
THC: ∆ ^9^-tetrahydrocannabinol

## Notes

Author Contributions M.J.B. led recruitment, designed the survey, analyzed the data, and wrote the manuscript. S.V.A. designed the survey, analyzed data, and assisted in manuscript write up. I.S.M. wrote and edited the manuscript and oversaw survey design. L.J.M. assisted in the statistical analysis of the data. A.S.S. was involved in survey design and HREC submissions. L.K.G. was involved in survey conception. C.J.C. and S.J.C. reviewed the manuscript and survey design. All authors reviewed and approved the final manuscript.

Funding: This work was supported by the Lambert Initiative for Cannabinoid Therapeutics, University of Sydney, NSW, Australia.

Conflicts of Interest: M.J.B., S.V.A., L.J.M., L.K.G., and A.S.S. have nothing to disclose. S.J.C. reports grants, personal fees, and other from Abbvie; other from Celgene; grants, personal fees, nonfinancial support, and other from Takeda; grants, personal fees, and other from Ferring; other from Gilead; grants, personal fees, and other from Janssen; grants and other from MSD; other from Novartis; grants, personal fees, nonfinancial support, and other from Pfizer; grants and personal fees from Shire; grants and nonfinancial support from Orphan; grants and nonfinancial support from Aspen; grants from Vifor; grants from South West Sydney Local Health District, outside the submitted work. C.J.C. reports personal fees from Abbvie, personal fees from Janssen, nonfinancial support from Takeda, outside the submitted work. I.S.M. reports personal fees from Janssen, other from Kinoxis Therapeutics, other from Canopy Growth Corporation, outside the submitted work. In addition, I.S.M. has patents AU2017904438, AU2017904072, and AU2018901971 pending.
