# Supplementary material for: Medicinal Cannabis for Inflammatory Bowel Disease: A Survey of Perspectives, Experiences, and Current Use in Australian Patients
Source: Crohns Colitis 360. 2020 Apr 16;2(2):otaa015. doi: 10.1093/crocol/otaa015 (PMC9802391; doi:10.1093/crocol/otaa015)
Supplement: otaa015_suppl_Supplementary_Tables [file otaa015_suppl_supplementary_tables.docx]

**Supplementary Tables**

**Supplementary Table S1:** Distribution of respondent recruitment sources

| ***How did you first hear about this survey?*** | **Non-users**  n (%) | **^#^All users**  n (%) |
| --- | --- | --- |
| Facebook | 318 (50.8%) | 114 (53.8%) |
| Twitter | 27 (4.3%) | 14 (6.6%) |
| Online Forum | 11 (1.8%) | 7 (3.3%) |
| A friend | 15 (2.4%) | 6 (2.8%) |
| Other | 11 (1.8%) | 5 (2.4%) |
| Consumer support Group | 8 (1.3%) | 2 (0.9%) |
| Other social media | 8 (1.3%) | 3 (1.4%) |
| Media | 3 (0.5%) | 2 (0.9%) |
| University Website | 3 (0.5%) | 1 (0.5%) |
| Doctor/healthcare provider | 1 (0.2%) | 0 (0%) |

*#All users column combines previous and current MC users.*

*Total valid responses for this survey item = 559/838. Number of respondents that were Non-users=405, All users= 154.*

**Supplementary Table S2:** Clinical characteristics of “IBD Unspecified” cohort

|  | | **IBD unspecified** | | |
| --- | --- | --- | --- | --- |
|  |  | **Non-users** | **^#^All users** | ***p value^a^*** |
| **BMI,** n (%) | Underweight (<18.5) | 0 (0%) | 1 (6.7%) | *^N/A^* |
|  | Healthy (18.5 to <25) | 4 (19%) | 8 (53.3%) | ***0.031*** |
|  | Overweight (25 to <30) | 9 (42.9%) | 5 (33.3%) | *0.56* |
|  | Obese (≥30) | 8 (38.1%) | 1 (6.7%) | ***0.032*** |
| **Disease years,** mean (SD) | | 8.76 (9.79) | 12.4 (12.24) | *0.33* |
| **Under the care of a GP for IBD**? n (% yes) | | 16 (80%) | 13 (93%) | *0.38* |
| **GP visits per year,** mean (SD) | | 9.88 (9.14) | 9.23 (8.89) | *0.89* |
| **Under the care of a specialist for IBD?** n (% yes) | | 13 (65%) | 7 (47%) | *0.32* |
| **Specialist visits per year,** mean (SD) | | 3.83 (3.95) | 2.86 (2.27) | *0.56* |
| **Current use of pharma drugs for IBD?** n (% yes) | | 10 (50%) | 5 (33%) | *0.49* |
| **MARS,** mean (SD) | | 7.33 (2.50) | 5.73 (2.40) | *0.063* |
| **Hospitalised for your IBD?** n (% yes) | | 12 (60%) | 7 (47%) | *0.51* |
| **# hospitalisations since diagnosis,** mean (SD) | | 5.0 (3.62) | 4.57 (2.7) | *0.79* |
| **Undergone surgery for IBD?** n (% yes) | | 6 (29%) | 2 (13%) | *0.42* |
| **SIBDQ,** mean (SD) | | 33.86 (10.6) | 31.8 (6.58) | *0.51* |
| **EQ-5D-5L, Utility score,** mean (SD) | | 0.460 (0.268) | 0.499 (0.212) | *0.64* |
| **EQ-5D-5L, Health today (out of 100),** mean (SD) | | 53.3 (25.7) | 60.6 (18.0) | *0.35* |
| **WPAI, Overall work impairment,** % (SD) | | 32.6% (40.4) | 34.8% (30.4) | *0.86* |
| **WPAI, Activity impairment,** % (SD) | | 48.1% (30.9) | 46.0% (21.3) | *0.82* |

*Number of respondents: IBDU non-users=20- 21, IBDU all users= 14-15*

*#All users column combines previous and current MC users.*

*^a^p value represents analysis of non-users vs all users for IBDU. Continuous variables were compared with Student’s t-test and discrete variables were compared with chi square analysis.*

*^b^BMI was compared using Pearson Chi-square test.*

*^N/A^ Chi-square analysis not able to be computed for comparisons as value <1.*

*IBDU; Inflammatory Bowel Disease Unspecified.SIBDQ; Short Inflammatory Bowel Disease Questionnaire. MARS: Medication Adherence Rating Scale. WPAI; Work Productivity and Activity Impairment Questionnaire. EQ-5D-5L; EuroQoL Five dimension Five level Questionnaire.*

**Supplementary Table S3:** Modified Disease Activity Indices for each IBD type

| **Disease-specific activity index** | | **Modifications** | **# items** | **Possible score range** | **Non-users** | **^#^All users** | ***p value**** |
| --- | --- | --- | --- | --- | --- | --- | --- |
| Crohn’s disease | **Partial CDAI,** mean (SD) | Removal of items 2,6,8 & 9:  (2) % below ideal body weight, (6) general wellbeing,  (8) abdominal mass  (9) hematocrit | 6 | 0-179 | 56.80 (34.12) | 59.46 (33.70) | *0.42* |
| Ulcerative Colitis | **Partial Mayo score,** mean (SD) | Removal of Item 4:  physician’s rating of disease activity | 3 | 0-9 | 3.4 (2.30) | 3.19 (2.33) | *0.57* |
| IBD Unspecified | **DAI,** mean (SD) | 4-point scale of IBD activity over past year:  Remission; in clinical remission (=0);  Mild disease activity; periods of remission with a few flare ups (=1);  Moderate disease activity; continuous mildly active disease (=2);  Severe disease activity; continuous significantly active disease (=3) | 1 | 0-3 | 1.7 (0.92) | 2.00 (0.66) | *0.29* |

*Number of respondents: CD non-users= 394, CD all users= 147; UC non-users= 211, UC all users= 49; IBDU non-users= 21, IBDU all users= 15.*

*#All users column combines previous and present MC users.*

**Analysis of all users (previous and present combined) versus non-users using a Student’s t-test.*

*CDAI: Crohn’s Disease Activity Index. DAI: Disease Activity Index.*
