# Supplementary material for: Medicinal Cannabis for Inflammatory Bowel Disease: A Survey of Perspectives, Experiences, and Current Use in Australian Patients
Source: Crohns Colitis 360. 2020 Apr 16;2(2):otaa015. doi: 10.1093/crocol/otaa015 (PMC9802391; doi:10.1093/crocol/otaa015)
Supplement: otaa015_suppl_Supplementary_Figure_S1 [file otaa015_suppl_supplementary_figure_s1.pdf]

Supplementary Figure 1

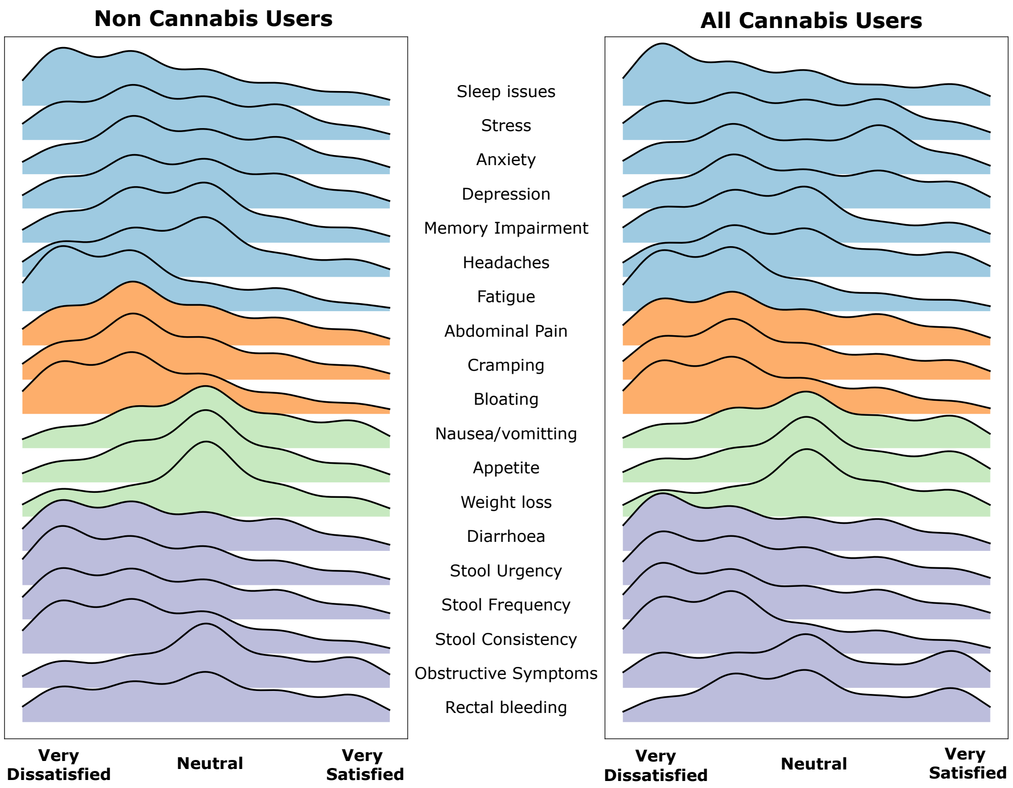

Supplementary Figure 1. Current symptom management satisfaction as reported by users (current and previous combined) and non-users. Respondent's overall satisfaction with symptom management was not different between cannabis use groups ($\chi2(4,2.587)$ p=0.62). Cannabis user respondents, n=205; non-user respondents, n=603.
