# Supplementary material for: Medicinal Cannabis for Inflammatory Bowel Disease: A Survey of Perspectives, Experiences, and Current Use in Australian Patients
Source: Crohns Colitis 360. 2020 Apr 16;2(2):otaa015. doi: 10.1093/crocol/otaa015 (PMC9802391; doi:10.1093/crocol/otaa015)
Supplement: otaa015_suppl_Supplementary_Figure_S2 [file otaa015_suppl_supplementary_figure_s2.pdf]

Supplementary Figure 2

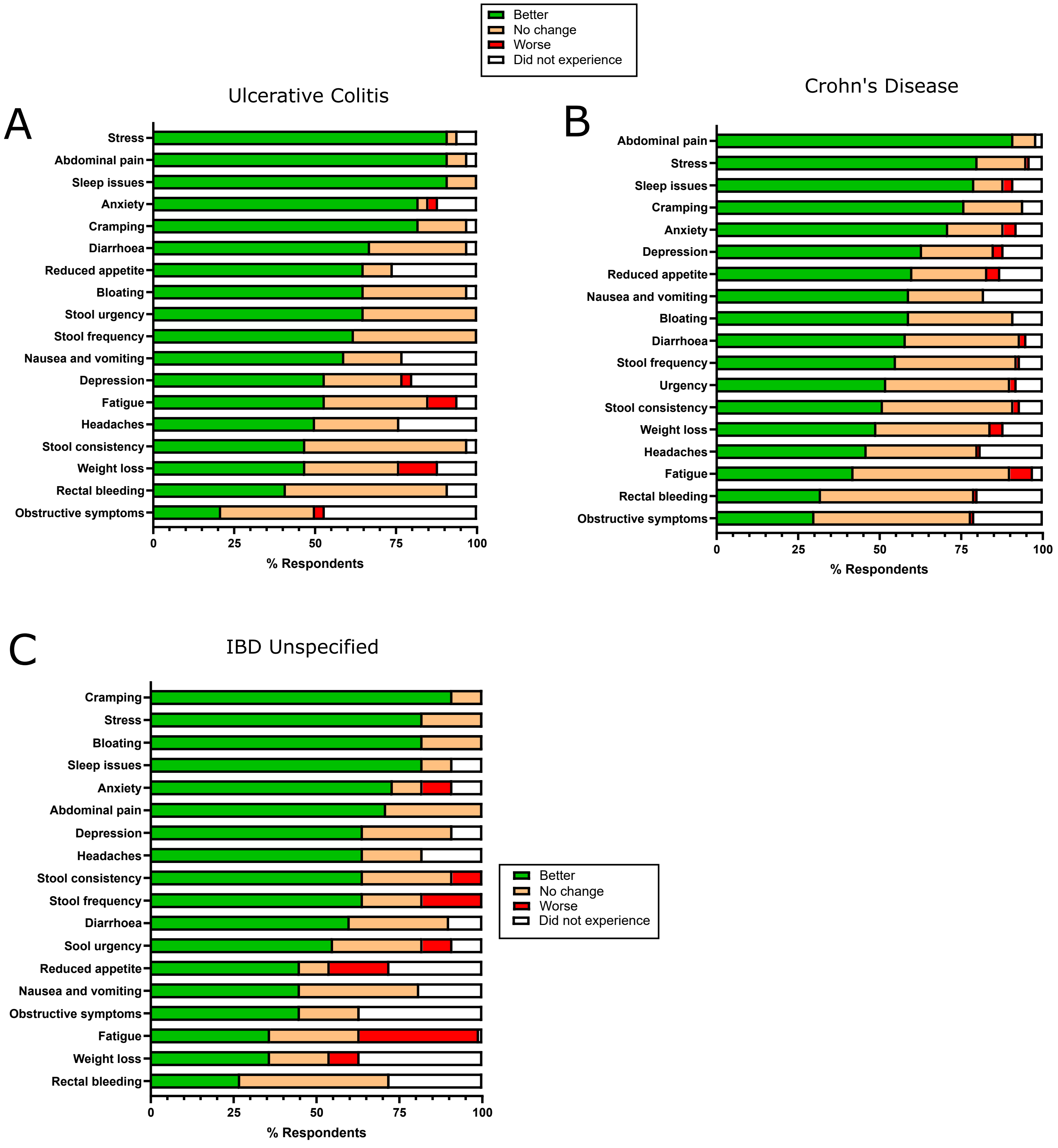

Supplementary Figure 2. Self-reported IBD symptom change with medicinal cannabis use in current and previous users listed by greatest positive benefit to least broken down by IBD type; **A**. Ulcerative colitis, **B**. Crohn's disease, **C**. IBD Unspecified. Green = positive change; orange = no change; red = negative change; white = did not report having this symptom.
