# Supplementary material for: Medicinal Cannabis for Inflammatory Bowel Disease: A Survey of Perspectives, Experiences, and Current Use in Australian Patients
Source: Crohns Colitis 360. 2020 Apr 16;2(2):otaa015. doi: 10.1093/crocol/otaa015 (PMC9802391; doi:10.1093/crocol/otaa015)
Supplement: otaa015_suppl_Supplementary_Material_REDCAP [file otaa015_suppl_supplementary_material_redcap.pdf]

Medicinal Cannabis for Inflammatory Bowel Disease: A Patient Survey of Perspectives and Experiences

As researchers from the University of Sydney's Lambert Initiative for Cannabinoid Therapeutics, we are launching an Australia wide online survey of people with inflammatory bowel disease that may currently use, have previously used or have never used medicinal cannabis for their IBD.

The survey will be anonymous and confidential and conducted through REDCap, a university based database that captures and secures your data confidentially. You will be allocated a user ID code that in no way allows for your responses to be matched to personal and/or identifying data.The survey will take between 10-20 minutes to complete and asks about:

1. You: your age, gender, level of education, current work and living conditions and your IBD;

2. How your IBD affects your day to day and symptoms you currently have;

3. How your IBD affects your work and productivity;

4. Your adherence to your prescribed IBD medications;

5. Your general health and quality of life;

6. Attitudes on medicinal cannabis and use preferences.

For further details and instructions please read the participant information statement attached here:

[Attachment: "IBD Patient Survey Participant Information Statement.pdf"]

Please indicate your eligibility for the study below:

- ☐ I am an Australian resident over the age of 18 who has been clinically diagnosed with Inflammatory Bowel Disease. Click here to confirm.
- ☐ I have read the Participant Information Statement and understand the nature of my involvement in this study. I understand I can withdraw my involvement at any time by exiting the server browser. In this case my data will not be saved or analysed. I also understand I can save my responses and return to complete this study at a later time. To do so I must record the User ID code provided when I select the "save and return" option at the end of any page. Click here to agree.

ABOUT YOU

What is your age (in years)?

____

Which gender do you identify with?

- ◯ Male
- ◯ Female
- ◯ Other

If chosen Other, please specify:

____

What is your height (cm)?

____

Question	Response
Which best describes your ethnicity?	◯ Indigenous Australian or Torres Strait Islander<br>◯ Caucasian/European descent<br>◯ Chinese<br>◯ Indian<br>◯ Middle Eastern<br>◯ Jewish<br>◯ Other
If chosen Other, please specify:	__________________________
What is your weight (kg)?	__________________________
Which state/territory of Australia do you live in?	◯ New South Wales<br>◯ Victoria<br>◯ Queensland<br>◯ Northern Territory<br>◯ Western Australia<br>◯ South Australia<br>◯ Tasmania<br>◯ Australian Capital Territory
What is the postcode of your current address?	__________________________
What is the highest level of education you have completed?	◯ Primary School<br>◯ Secondary School<br>◯ Trade or Vocational<br>◯ University Degree<br>◯ Other
If chosen Other, please specify:	__________________________
Are you currently employed?	◯ No<br>◯ Yes
Which option best describes your current employment status?	◯ Full-time<br>◯ Part-time<br>◯ Home duties<br>◯ Unemployed<br>◯ Retired<br>◯ Disability Pension<br>◯ Student
What is your current relationship status?	◯ Single<br>◯ Partnered
Do you currently smoke cigarettes (tobacco)?	◯ No<br>◯ Yes<br>◯ Previously but now stopped

ABOUT YOUR IBD

Which inflammatory bowel disease (IBD) have you been diagnosed with?	◯ Ulcerative Colitis<br>◯ Crohn's Disease<br>◯ IBD unspecified<br>◯ Other<br>◯ I do not have IBD
If chosen Other, please specify your IBD diagnosis:	__________________________
During the past week, my stool frequency has been:	◯ Normal<br>◯ 1-2 stools/day more than normal<br>◯ 3-4 stools/day more than normal<br>◯ >4 stools/day more than normal
During the past week, I have experienced rectal bleeding:	◯ None of the time<br>◯ Visible blood with stool less than half the time<br>◯ Visible blood with stool half the time or more
Based on the appearance of my bowels following endoscopy, I have:	◯ Normal or inactive disease<br>◯ Mild disease (erythema, decreased vascular pattern, mild friability)<br>◯ Moderate disease (marked erythema, absent ascular pattern, friability, erosions)<br>◯ Severe disease (spontaneous bleeding, ulceration)<br>◯ Unsure<br>(Erythema=redness of the skin or mucous membrane | Friability=inflammation or ease of bleeding on the mucous membrane)
Over the past week, abdominal pain has been:	◯ None<br>◯ Mild<br>◯ Moderate<br>◯ Severe
Number of soft/liquid stools in the last 7 days:	◯ None<br>◯ 1-2 stools<br>◯ 3-5 stools<br>◯ >6 stools
I have used anti-diarrhoea medication during the last week:	◯ Yes<br>◯ No
I experience the following extra-intestinal complications:	☐ None<br>☐ Arthritis/arthralgias (i.e. joint pain or discomfort)<br>☐ Iritis/uveitis (i.e. inflammation of the eye)<br>☐ Erythema nodosum (i.e. tender red nodules or lumps on the shins, thighs or forearms)<br>☐ Pyoderma gangrenosum (i.e. large sores/ulcers on the skin, often the legs)<br>☐ Apthous stomatitis (i.e. canker sores or mouth ulcers)<br>☐ Anal fissure, fistula or abscess<br>☐ Other fistula<br>☐ Fever/temperature >37.8C<br>(Select all that apply)

Which of the following best describes the disease activity of your IBD over the past year?	◯ Remission: in clinical remission<br>◯ Mild disease activity: Periods of remission with a few flare ups<br>◯ Moderate disease activity: Continuous mildly active disease<br>◯ Severe disease activity: Continuous significantly active disease
At what age were you diagnosed with your IBD?	__________________________________
Have you ever been hospitalised for complications relating to your IBD?	◯ Yes<br>◯ No
If yes, how many times have you been hospitalised for your IBD in your lifetime?	◯ Never<br>◯ 1-3 times<br>◯ 4-6 times<br>◯ 6-10 times<br>◯ >10 times
Have you undergone surgery for your IBD?	◯ Yes<br>◯ No
If yes, how did the surgery affect the disease activity of your IBD once recovered from surgery?	◯ Significant worsening<br>◯ Slight worsening<br>◯ No change<br>◯ Slight improvement<br>◯ Significant improvement

You have completed 1 of 6 pages so far.

Please click the ONE box that best describes your health TODAY.

1) MOBILITY

- ◯ I have no problems with walking around
- ◯ I have slight problems with walking around
- ◯ I have moderate problems with walking around
- ◯ I have severe problems with walking around
- ◯ I am unable to walk around

Please click the ONE box that best describes your health TODAY.

2) PERSONAL CARE

- ◯ I have no problems with washing or dressing myself
- ◯ I have slight problems with washing or dressing myself
- ◯ I have moderate problems with washing or dressing myself
- ◯ I have severe problems with washing or dressing myself
- ◯ I am unable to wash or dress myself

Please click the ONE box that best describes your health TODAY.

3) USUAL ACTIVITIES (e.g. work, study, housework, family or leisure activities)

- ◯ I have no problems doing my usual activities
- ◯ I have slight problems doing my usual activities
- ◯ I have moderate problems doing my usual activities
- ◯ I have severe problems doing my usual activities
- ◯ I am unable to do my usual activities

Please click the ONE box that best describes your health TODAY.

4) PAIN / DISCOMFORT

- ◯ I have no pain or discomfort
- ◯ I have slight pain or discomfort
- ◯ I have moderate pain or discomfort
- ◯ I have severe pain or discomfort
- ◯ I have extreme pain or discomfort

Please click the ONE box that best describes your health TODAY.

5) ANXIETY / DEPRESSION

- ◯ I am not anxious or depressed
- ◯ I am slightly anxious or depressed
- ◯ I am moderately anxious or depressed
- ◯ I am severely anxious or depressed
- ◯ I am extremely anxious or depressed

6)

We would like to know how good or bad your health is TODAY.

This scale is numbered from 0 to 100.

100 means the best health you can imagine.
0 means the worst health you can imagine.
Please click on the scale to indicate how your health is TODAY.

<strong>
0 - The worst health you can imagine <strong>50 <strong>100 - The best health you can imagine

(Place a mark on the scale above)

EuroQol Research Foundation. EQ-5D is a trade mark of the EuroQol Research Foundation

You have completed 2 of 6 pages so far.

Are you currently taking any pharmaceutical drugs to manage your IBD or related symptoms?

◯ Yes
◯ No
(e.g. corticosteroids, aminosalicylates, immunomodulators, biologic agents, analgesics, antibiotics, antidepressants)

If yes, which of the following pharmaceutical medications are you currently taking to manage your IBD? How would you rate your overall satisfaction with these treatments?

	Very satisfied	Somewhat satisfied	Neutral	Somewhat dissatisfied	Very dissatisfied	N/A
Corticosteroids (e.g. prednisone)	◯	◯	◯	◯	◯	◯
Aminosalicylates (e.g. sulfasalazine, mesalamine, olsalazine, balsalazide)	◯	◯	◯	◯	◯	◯
Immunomodulators (e.g. azathioprine, 6-mercaptopurine, tacrolimus, methotrexate)	◯	◯	◯	◯	◯	◯
Calcineurin inhibitors (e.g. cyclosporine, tacrolimus)	◯	◯	◯	◯	◯	◯
Biologic agents (e.g. infliximab, adalimumab, vedolizumab, ustekinumab)	◯	◯	◯	◯	◯	◯
Analgesics (e.g. codeine, oxycodone, tramadol, ibuprofen, Panadol)	◯	◯	◯	◯	◯	◯
Antibiotics (e.g. ciprofloxacin, metronidazole)	◯	◯	◯	◯	◯	◯
Antidepressants/ anti-anxiety: (e.g. SSRIs, SNRIs, tricyclics)	◯	◯	◯	◯	◯	◯
Benzodiazepines: (Diazepam, alprazolam)	◯	◯	◯	◯	◯	◯

PROFESSIONAL CARE

I am under the care of a general practitioner

◯ Yes
◯ No

If yes, on average, how often do you visit your general practitioner for you IBD?

◯ 2 visits per year (every 6 months)
◯ 4 visits per year (every 3 months)
◯ 8 visits per year (every 6 weeks)
◯ 16 visits per year (every 3 weeks)
◯ >25 visits per year (nearly every fortnight)

I am under the care of a gastroenterological specialist

◯ Yes
◯ No

If yes, how often do you see your gastroenterologist?

- ◯ 2 visits per year (every 6 months)
- ◯ 4 visits per year (every 3 months)
- ◯ 8 visits per year (every 6 weeks)
- ◯ 16 visits per year (every 3 weeks)
- ◯ >25 visits per year (nearly every fortnight)

ALTERNATIVE/COMPLIMENTARY THERAPIES

Do you currently use any of the following alternative/complementary therapies or supplements to manage your IBD?

- ☐ Prebiotics or Probiotics
- ☐ Fish oil (omega-3 fatty acid) supplement
- ☐ Herbal or dietary supplments
- ☐ Restriction/exclusion diety (e.g. low FODMAP diet)
- ☐ Stress management (e.g. cognitive behavioural therapy, mindfulness meditation)
- ☐ Faecal microbiota transplantation (FMT)
- ☐ Traditional Chinese medicine/acupuncture or moxibustion
- ☐ Chiropractic/osteopathy/massage
- ☐ Cannabis
- ☐ Gut-directed hypnotherapy
- ☐ None of the above
- ☐ Other

(Select all that apply)

If answered Other, please specify: ______________________________

How would you rate your overall satisfaction in management of the below symptoms relating to your IBD in the past year?

	Very satisfied	Somewhat Satisfied	Neutral	Somewhat Dissatisfied	Very Dissatisfied	I do not have this symptom
Anxiety	◯	◯	◯	◯	◯	◯
Stress	◯	◯	◯	◯	◯	◯
Depression	◯	◯	◯	◯	◯	◯
Memory impairment	◯	◯	◯	◯	◯	◯
Fatigue	◯	◯	◯	◯	◯	◯
Headaches	◯	◯	◯	◯	◯	◯
Appetite	◯	◯	◯	◯	◯	◯
Nausea/vomiting	◯	◯	◯	◯	◯	◯
Diarrhoea	◯	◯	◯	◯	◯	◯
Abdominal pain	◯	◯	◯	◯	◯	◯
Bloating	◯	◯	◯	◯	◯	◯
Cramping	◯	◯	◯	◯	◯	◯
Stool consistency	◯	◯	◯	◯	◯	◯
Stool frequency	◯	◯	◯	◯	◯	◯
Stool urgency	◯	◯	◯	◯	◯	◯

Obstructive symptoms (fistula, abscess, stricture)	○	○	○	○	○	○
Rectal bleeding	○	○	○	○	○	○
Weight loss	○	○	○	○	○	○
Sleep issues	○	○	○	○	○	○

You have completed 3 of 6 pages so far.

INSTRUCTIONS FOR SELF-COMPLETED SIBDQ

This questionnaire is designed to measure the effects of your inflammatory bowel disease on your daily function and quality of life. You will be asked about symptoms you have been having as a result of your bowel disease, the way you have been feeling in general, and how your mood has been.

On this questionnaire there are 10 questions. Each question has a graded response numbered from 1 to 7. Please read each question carefully and select the number which best describes how you have been feeling in the past 2 weeks.

EXAMPLE

How often have you felt unwell as a result of your bowel problem in the past 2 weeks?

1 ALWAYS
2 ALMOST ALWAYS
3 MANY TIMES
4 SOMETIMES
5 RARELY
6 VERY RARELY
7 NEVER

If you are having trouble understanding a question, STOP for a moment! Think about what the question means to you. How is this activity or issue affected by your bowel problem? Then answer the question as best as you can. You will have the chance to ask the research assistant questions after completing the questionnaire. This only takes a few minutes to complete.

Copyright ©1989 McMaster University, Hamilton, Ontario, Canada

The Inflammatory Bowel Disease Questionnaire (IBDQ), authored by Dr. Jan Irvine et al, is the copyright of McMaster University (Copyright ©1989, McMaster University). The IBDQ has been provided under licence from McMaster University and must not be copied, distributed or used in any way without the prior written consent of McMaster University.

Contact the McMaster Industry Liaison Office at McMaster University, email: milo@mcmaster.ca for licencing details.

QUALITY OF LIFE IN SHORT INFLAMMATORY BOWEL DISEASE QUESTIONNAIRE (SIBDQ)

This questionnaire is designed to find out how you have been feeling during the last 2 weeks. You will be asked about symptoms you have been having as a result of your inflammatory bowel disease, the way you have been feeling in general, and how your mood has been.

1. How often has the feeling of fatigue or of being tired and worn out been a problem for you during the last 2 weeks? Please indicate how often the feeling of fatigue or tiredness has been a problem for you during the last 2 weeks by picking one of the options from	◯ 1 ALWAYS<br>◯ 2 ALMOST ALWAYS<br>◯ 3 MANY TIMES<br>◯ 4 SOMETIMES<br>◯ 5 RARELY<br>◯ 6 VERY RARELY<br>◯ 7 NEVER

2. How often during the last 2 weeks have you had to delay or cancel a social engagement because of your bowel problem? Please choose an option from	◯ 1 ALWAYS<br>◯ 2 ALMOST ALWAYS<br>◯ 3 MANY TIMES<br>◯ 4 SOMETIMES<br>◯ 5 RARELY<br>◯ 6 VERY RARELY<br>◯ 7 NEVER
3. How much difficulty have you had, as a result of your bowel problems, doing leisure or sports activities you would have liked to have done during the last 2 weeks? Please choose an option from	◯ 1 AN EXTREME AMOUNT OF DIFFICULTY; ACTIVITIES MAD IMPOSSIBLE<br>◯ 2 A LOT OF DIFFICULTY<br>◯ 3 A FAIR BIT OF DIFFICULTY<br>◯ 4 AN AVERAGE AMOUNT OF DIFFICULTY<br>◯ 5 A LITTLE DIFFICULTY<br>◯ 6 HARDLY ANY DIFFICULTY<br>◯ 7 NO DIFFICULTY; THE BOWEL PROBLEMS DID NOT LIMIT SPORTS OR LEISURE ACTIVITIES AT ALL
4. How often during the last 2 weeks have you been troubled by pain in the abdomen? Please choose an option from	◯ 1 ALWAYS<br>◯ 2 ALMOST ALWAYS<br>◯ 3 MANY TIMES<br>◯ 4 SOMETIMES<br>◯ 5 RARELY<br>◯ 6 VERY RARELY<br>◯ 7 NEVER
5. How often during the last 2 weeks have you felt depressed or discouraged? Please choose an option from	◯ 1 ALWAYS<br>◯ 2 ALMOST ALWAYS<br>◯ 3 MANY TIMES<br>◯ 4 SOMETIMES<br>◯ 5 RARELY<br>◯ 6 VERY RARELY<br>◯ 7 NEVER
6. Overall, in the last 2 weeks, how much of a problem have you had with passing large amounts of gas? Please choose an option from	◯ 1 EXTREMELY PROBLEMATIC<br>◯ 2 VERY PROBLEMATIC<br>◯ 3 SOMEWHAT PROBLEMATIC<br>◯ 4 SLIGHTLY PROBLEMATIC<br>◯ 5 VERY SLIGHTLY PROBLEMATIC<br>◯ 6 ALMOST NOT PROBLEMATIC<br>◯ 7 NOT PROBLEMATIC
7. Overall, in the last 2 weeks, how much of a problem have you had maintaining or getting to, the weight you would like to be at? Please choose an option from	◯ 1 EXTREMELY PROBLEMATIC<br>◯ 2 VERY PROBLEMATIC<br>◯ 3 SOMEWHAT PROBLEMATIC<br>◯ 4 SLIGHTLY PROBLEMATIC<br>◯ 5 VERY SLIGHTLY PROBLEMATIC<br>◯ 6 ALMOST NOT PROBLEMATIC<br>◯ 7 NOT PROBLEMATIC
8. How often during the last 2 weeks have you felt relaxed and free of tension? Please choose an option from	◯ 1 NEVER<br>◯ 2 VERY RARELY<br>◯ 3 RARELY<br>◯ 4 SOMETIMES<br>◯ 5 MANY TIMES<br>◯ 6 ALMOST ALWAYS<br>◯ 7 ALWAYS

9. How much of the time during the last 2 weeks have you been troubled by a feeling of having to go to the toilet even though your bowels were empty? Please choose an option from	◯ 1 ALWAYS<br>◯ 2 ALMOST ALWAYS<br>◯ 3 MANY TIMES<br>◯ 4 SOMETIMES<br>◯ 5 RARELY<br>◯ 6 VERY RARELY<br>◯ 7 NEVER
10. How much of the time during the last 2 weeks have you felt angry as a result of your bowel problem? Please choose an option from	◯ 1 ALWAYS<br>◯ 2 ALMOST ALWAYS<br>◯ 3 MANY TIMES<br>◯ 4 SOMETIMES<br>◯ 5 RARELY<br>◯ 6 VERY RARELY<br>◯ 7 NEVER

Work Productivity and Activity Impairment Questionnaire (WPAI).

The following questions ask about the effect of your IBD on your ability to work and perform regular activities.

Are you currently employed (working for pay)?	◯ Yes<br>◯ No
The next questions are about the past seven days, not including today.	
During the past seven days, how many hours did you miss from work because of your IBD?<br><br>Include hours you missed on sick days, times you went in late, left early, etc., because of your health problems. Do not include time you missed to participate in this study.	________________________
During the past seven days, how many hours did you miss from work because of any other reason, such as vacation, holidays, time off to participate in this study?	________________________
During the past seven days, how many hours did you actually work?	________________________
During the past 7 days, how much did your IBD affect your productivity while you were working?<br><br>Think about how many days you were limited in the amount or kind of work you could do, days you accomplished less than you would like or days you could not do your work as carefully as usual. If your IBD affected your work only a little, choose a low number. Choose a high number if your IBD affected your work a great deal.	◯ 0 (IBD had no effect on work)<br>◯ 1<br>◯ 2<br>◯ 3<br>◯ 4<br>◯ 5<br>◯ 6<br>◯ 7<br>◯ 8<br>◯ 9<br>◯ 10 (IBD completely prevented me from working)

During the past 7 days, how much did your IBD affect your ability to do your regular daily activities other than work at a job?

By regular activities, we mean the usual activities you do, such as work around the house, shopping, child care, exercising, studying etc. Think about times you were limited in the amount or kind of activities you could do and times you accomplished less than you would like. If your IBD affected your activities only a little, choose a low number. If your IBD affected your activities a lot, choose a high number.

- ◯ 0 (IBD had no effect on my daily activities)
- ◯ 1
- ◯ 2
- ◯ 3
- ◯ 4
- ◯ 5
- ◯ 6
- ◯ 7
- ◯ 8
- ◯ 9
- ◯ 10 (IBD completely prevented me from doing my daily activities)

You have completed 4 of 6 pages so far.

Medication Adherence Rating Scale (MARS).

This series of questions helps to determine your adherence to medications, not including medicinal cannabis. This may refer to any medications that are prescribed for your IBD by your doctor.

1)	Do you ever forget to take your medication?	◯ Yes<br>◯ No
2)	Are you careless at times about taking your medication?	◯ Yes<br>◯ No
3)	When you feel better, do you sometimes stop taking your medication?	◯ Yes<br>◯ No
4)	Sometimes I you feel worse when you take the medication, do you stop taking it?	◯ Yes<br>◯ No
5)	I take my medication only when I'm sick	◯ Yes<br>◯ No
6)	It is unnatural for my mind and body to be controlled by medication	◯ Yes<br>◯ No
7)	My thoughts are clearer on medication	◯ Yes<br>◯ No
8)	By staying on medication, I can prevent getting sick	◯ Yes<br>◯ No
9)	I feel weird, like a "zombie" on medication	◯ Yes<br>◯ No
10)	Medication makes me feel tired and sluggish	◯ Yes<br>◯ No

You have completed 5 of 6 pages so far.

CANNABIS USE FOR IBD SYMPTOMS

Question	Options
Have you ever used cannabis to manage your IBD symptoms?	◯ Yes, I currently use cannabis to manage my IBD symptoms<br>◯ Yes, I have previously used cannabis to manage my IBD symptoms but I have now stopped<br>◯ No, I have never used cannabis to manage my IBD symptoms
In your opinion, what should be the legal status of cannabis use in Australia?	◯ Cannabis use should be legal for ALL purposes (recreational and medicinal)<br>◯ Cannabis use should be legal for medicinal purposes only<br>◯ Cannabis should be illegal for all purposes<br>◯ Unsure<br>◯ No opinion

PRESENT USERS

Attitudes on medicinal cannabis and use preferences of present users. You indicated you are currently using medicinal cannabis for your IBD. This section will ask about your current use as well as you attitudes and experiences with medicinal cannabis and your IBD.

Question	Options
Which of the following informed your decision to use medicinal cannabis for your IBD?	◯ Discovered benefits on my own internet-based media<br>◯ Conventional media<br>◯ Conventional healthcare provider<br>◯ Alternative healthcare practitioner<br>◯ Friend or family member<br>◯ Medicinal cannabis advocacy group<br>◯ Other
How frequently do you use cannabis medicinally to treat symptoms relating to your IBD?	◯ Multiple times a day<br>◯ Every day<br>◯ Several times a week<br>◯ Once a week<br>◯ Several times a month<br>◯ Once a month

What is the main way you consume cannabis for medicinal purposes?	◯ Oral (tablet or capsule)<br>◯ Oral edibles (cake, cookie)<br>◯ Oral liquid (oil, tincture)<br>◯ Oral spray (mouth or oromucosal)<br>◯ Fresh juice<br>◯ Nasal application (through nose)<br>◯ Rolled into a joint<br>◯ Pipe (plastic, metal, glass)<br>◯ Water pipe/bong<br>◯ Suppository<br>◯ Dabbing or spotting<br>◯ Topical (cream/patch applied to the skin)<br>◯ Vaporizer<br>◯ Other<br>(Please only select one option)
If you answered Other, which other ways do you consume medicinal cannabis?	__________________________________
What type of medicinal cannabis do you mainly use?	◯ THC only<br>◯ Mainly THC and small amounts of other cannabinoids<br>◯ Approximately equal amounts of THC and CBD<br>◯ Mainly CBD and small amounts of other cannabinoids (e.g. THC)<br>◯ CBD only<br>◯ Unsure/Do not know<br>◯ It varies between batches<br>◯ Other<br>(Please only select one option)
If you answered Other, which other type of cannabis do you mainly use?	__________________________________
What is the main way you access your medicinal cannabis?	◯ Cannabis access clinic<br>◯ I grow my own<br>◯ From friends or family<br>◯ From a medicinal cannabis supplier (not prescribed by a medical practitioner)<br>◯ Prescribed by a medical practitioner and dispensed from a pharmacy<br>◯ From a club or co-operative outlet<br>◯ From an online supplier<br>◯ From an overseas supplier<br>◯ From a recreational dealer<br>◯ Other<br>(Please only select one option)
If you answered Other, which other ways do you access cannabis?	__________________________________
In the last 28 days, have you ever been unable to access medicinal cannabis to treat your IBD?	◯ Yes<br>◯ No
Do you worry about accessing your preferred source of cannabis?	◯ Very much so<br>◯ Somewhat<br>◯ No opinion,<br>◯ Not especially<br>◯ Not at all

Do you consider medicinal cannabis successful in managing your IBD symptoms?	◯ Yes<br>◯ No
Which option best describes how your IBD compares now to how it was before using medicinal cannabis?	◯ Very much better<br>◯ Much better<br>◯ A little better<br>◯ No change<br>◯ A little worse<br>◯ Much worse<br>◯ Very much worse

Which of the following symptoms were altered following medicinal cannabis use and how did this symptom change with medicinal cannabis use?

	Very much better	Much better	A little better	No change	A little worse	Very much worse	I do not have this symptom
Anxiety	◯	◯	◯	◯	◯	◯	◯
Stress	◯	◯	◯	◯	◯	◯	◯
Depression	◯	◯	◯	◯	◯	◯	◯
Memory impairment	◯	◯	◯	◯	◯	◯	◯
Fatigue	◯	◯	◯	◯	◯	◯	◯
Headaches	◯	◯	◯	◯	◯	◯	◯
Reduced appetite	◯	◯	◯	◯	◯	◯	◯
Nausea/vomiting	◯	◯	◯	◯	◯	◯	◯
Diarrhoea	◯	◯	◯	◯	◯	◯	◯
Abdominal pain	◯	◯	◯	◯	◯	◯	◯
Bloating	◯	◯	◯	◯	◯	◯	◯
Cramping	◯	◯	◯	◯	◯	◯	◯
Stool consistency	◯	◯	◯	◯	◯	◯	◯
Stool frequency	◯	◯	◯	◯	◯	◯	◯
Urgency	◯	◯	◯	◯	◯	◯	◯
Obstructive symptoms (fistula, abscess, stricture)	◯	◯	◯	◯	◯	◯	◯
Rectal bleeding	◯	◯	◯	◯	◯	◯	◯
Weight loss	◯	◯	◯	◯	◯	◯	◯
Sleep issues	◯	◯	◯	◯	◯	◯	◯

Were any other symptoms altered following medicinal cannabis use?	◯ Yes<br>◯ No
Describe the other symptoms which improved following medicinal cannabis use	__________________________________

What effect did medicinal cannabis have on this symptom?

◯ Very much better
◯ Much better
◯ A little better
◯ No change
◯ A little worse
◯ Much worse
◯ Very much worse

Side effects

Indicate whether you have experienced any of the following side effects in the last 12 months when using medicinal cannabis, and, if so, how serious these side effects were.

Note: "mild and tolerable" = symptoms that caused only mind impairment of function and do not require specific treatment (e.g. other medications or stopping medicinal cannabis); "Severe and intolerable" = symptoms that caused impairment of function and/or resulted in either other treatment (e.g. other medications) or resulted in stopping medicinal cannabis use.

	Did not experience	Mild and tolerable	Severe and intolerable
Allergies	◯	◯	◯
Anxiety	◯	◯	◯
Cannabis hyperemesis	◯	◯	◯
Confusion	◯	◯	◯
Constipation	◯	◯	◯
Decreased appetite	◯	◯	◯
Dehydration	◯	◯	◯
Delusions	◯	◯	◯
Depressed mood	◯	◯	◯
Diarrhoea	◯	◯	◯
Dry mouth	◯	◯	◯
Dizziness	◯	◯	◯
Drowsiness or sedation	◯	◯	◯
Eye Irritation	◯	◯	◯
Gastrointestinal irritation	◯	◯	◯
Hallucinations	◯	◯	◯
Headaches	◯	◯	◯
Increased appetite	◯	◯	◯
Lack of energy or fatigue	◯	◯	◯
Memory impairment	◯	◯	◯
Nasopharyngeal complaints	◯	◯	◯
Nausea and/or vomitting	◯	◯	◯
Panic attack	◯	◯	◯

Paranoia	○	○	○
Racing heart/palpitations	○	○	○
Bad taste in mouth	○	○	○
Respiratory complaints	○	○	○
Shaking/tremor/poor movement control	○	○	○
Sleep disturbance	○	○	○
Sweating	○	○	○

Which of the following best describes how your use of each drug changed whilst using medicinal cannabis to treat your symptoms of IBD?

Note: A moderate change = more than 20% difference, a marked change is more than 50% change (e.g. a marked decrease would be reducing the drug by more than half).

	Markedly decreased	Moderately decreased	No change	Moderately increased	Markedly increased	I do not take this medication
Corticosteroids (e.g. prednisone)	○	○	○	○	○	○
Aminosalicylates (e.g. sulfasalazine, mesalamine, olsalazine, balsalazide)	○	○	○	○	○	○
Immunomodulators (e.g. azathioprine, 6-mercaptopurine, tacrolimus, methotrexate)	○	○	○	○	○	○
Calcineurin inhibitors (e.g. cyclosporine, tacrolimus)	○	○	○	○	○	○
Biologic agents (e.g. infliximab, adalimumab, vedolixumab, ustekinumab)	○	○	○	○	○	○
Analgesics (e.g. codeine, oxycodone, tramadol, ibuprofen, Panadol)	○	○	○	○	○	○
Antibiotics (e.g. ciprofloxacin, metronidazole)	○	○	○	○	○	○
Antidepressants/anti-anxiety: (e.g. SSRIs, SNRIs, tricyclics)	○	○	○	○	○	○
Benzodiazepines: (Diazepam, alprazolam)	○	○	○	○	○	○

Are you currently also using cannabis for non-medical purposes (recreationally)?

- ○ Yes
- ○ No

If yes, how frequently do you use cannabis for non-medical purposes (recreationally)?

- ○ Multiple times a day
- ○ Every day
- ○ Several times a week
- ○ Once a week
- ○ Several times a month
- ○ Once a month

PAST USERS

Attitudes on medicinal cannabis and use preferences of previous but not current users.You previously indicated you have used medicinal cannabis in the past but do not currently use.

This section will ask about your previous use as well as you attitudes and past experiences with medicinal cannabis and your IBD.

Which of the following informed your decision to use medicinal cannabis for your IBD?

- ◯ Discovered benefits on my own Internet-based media
- ◯ Conventional media
- ◯ Conventional healthcare provider
- ◯ Alternative healthcare practitioner
- ◯ Friend or family member
- ◯ Medicinal cannabis advocacy group
- ◯ Other

If chosen Other, please specify:

____

How frequently did you use cannabis medicinally to treat symptoms relating to your IBD?

- ◯ Multiple times a day
- ◯ Every day
- ◯ Several times a week
- ◯ Once a week
- ◯ Several times a month
- ◯ Once a month

What was the main way you consumed cannabis for medicinal purposes?

- ◯ Oral (tablet or capsule)
- ◯ Oral edibles (cake, cookie)
- ◯ Oral liquid (tincture, etc.)
- ◯ Oral spray (mouth or oromucosal)
- ◯ Fresh juice
- ◯ Nasal application (through nose)
- ◯ Rolled into a joint
- ◯ Pipe (plastic, metal, glass)
- ◯ Water pipe/bong
- ◯ Suppository
- ◯ Dabbing or spotting
- ◯ Topical (cream/patch applied to the skin)
- ◯ Vaporizer
- ◯ Other

(Please only select one option)

If you answered Other, which other type of cannabis did you mainly use?

____

What is the main way you accessed your medicinal cannabis?

- ◯ Cannabis access clinic
- ◯ I grow my own
- ◯ From friends or family
- ◯ From a medicinal cannabis supplier (not prescribed by a medical practitioner)
- ◯ Prescribed by a medical practitioner and dispensed from a pharmacy
- ◯ From a club or co-operative outlet
- ◯ From an online supplier
- ◯ From an overseas supplier
- ◯ From a recreational dealer
- ◯ Other

(Please only select one option)

If you answered Other, which other ways did you access cannabis?	__________________________
Have you ever experienced difficulties with accessing medicinal cannabis to treat your IBD?	◯ Yes<br>◯ No
Did you worry about accessing your preferred source of cannabis?	◯ Very much so<br>◯ Somewhat<br>◯ No opinion,<br>◯ Not especially<br>◯ Not at all
Did you consider medicinal cannabis successful in managing your IBD symptoms?	◯ Yes<br>◯ No
Which option best describes how your IBD compares now to how it was before using medicinal cannabis?	◯ Very much better<br>◯ Much better<br>◯ A little better<br>◯ No change<br>◯ A little worse<br>◯ Much worse<br>◯ Very much worse

Which of the following symptoms were altered following medicinal cannabis use and how did this symptom change with medicinal cannabis use?

	Very much better	A little better	No change	A little worse	Much worse	Very much worse	I do not have this symptom
Anxiety	◯	◯	◯	◯	◯	◯	◯
Stress	◯	◯	◯	◯	◯	◯	◯
Depression	◯	◯	◯	◯	◯	◯	◯
Memory impairment	◯	◯	◯	◯	◯	◯	◯
Fatigue	◯	◯	◯	◯	◯	◯	◯
Headaches	◯	◯	◯	◯	◯	◯	◯
Reduced appetite	◯	◯	◯	◯	◯	◯	◯
Nausea/vomiting	◯	◯	◯	◯	◯	◯	◯
Diarrhoea	◯	◯	◯	◯	◯	◯	◯
Abdominal pain	◯	◯	◯	◯	◯	◯	◯
Bloating	◯	◯	◯	◯	◯	◯	◯
Cramping	◯	◯	◯	◯	◯	◯	◯
Stool consistency	◯	◯	◯	◯	◯	◯	◯
Stool frequency	◯	◯	◯	◯	◯	◯	◯
Urgency	◯	◯	◯	◯	◯	◯	◯
Obstructive symptoms (fistula, abscess, stricture)	◯	◯	◯	◯	◯	◯	◯

Rectal bleeding	○	○	○	○	○	○	○
Weight loss	○	○	○	○	○	○	○
Sleep issues	○	○	○	○	○	○	○

Side effects

Indicate whether you experienced any of the following side effects when using medicinal cannabis, and, if so, how serious these side effects were.

Note: "Mild and intolerable" = symptoms that caused only mild impairment of function and do not require specific treatment (e.g. other medications or stopping medicinal cannabis); "Severe or intolerable" = symptoms that caused impairment of function and/or resulted in either other treatment (e.g. other medications) or resulted in stopping medicinal cannabis use.

	I did not experience this side effect	Mild and tolerable	Severe and intolerable
Allergies	○	○	○
Anxiety	○	○	○
Cannabis hyperemesis	○	○	○
Confusion	○	○	○
Constipation	○	○	○
Decreased appetite	○	○	○
Dehydration	○	○	○
Delusions	○	○	○
Depressed mood	○	○	○
Diarrhoea	○	○	○
Dry mouth	○	○	○
Dizziness	○	○	○
Drowsiness or sedation	○	○	○
Eye irritation	○	○	○
Gastrointestinal irritation	○	○	○
Hallucinations	○	○	○
Headaches	○	○	○
Increased appetite	○	○	○
Lack of energy or fatigue	○	○	○
Memory impairment	○	○	○
Nasopharyngeal complaints	○	○	○
Nausea and/or vomiting	○	○	○
Panic attack	○	○	○
Paranoia	○	○	○

Racing heart/palpitations	◯	◯	◯
Bad taste in mouth	◯	◯	◯
Respiratory complaints	◯	◯	◯
Shaking, tremors, poor movement control	◯	◯	◯
Sleep disturbance	◯	◯	◯
Sweating	◯	◯	◯

Why did you discontinue your use of medicinal cannabis for treatment of symptoms relating to your IBD?

- ◯ It was too expensive
- ◯ I had difficulties buying or accessing it
- ◯ Advice of my health provider (e.g. GP, medical specialist)
- ◯ I was concerned about legal consequences of using an illicit drug
- ◯ I was concerned about on-road random drug testing (RDT)
- ◯ My cannabis use interfered with my job/social life
- ◯ Other

If chosen Other, please specify: ______________________

Would you be interested in using medicinal cannabis in the future to treat symptoms relating to your IBD?

- ◯ Yes
- ◯ No

NEVER USED

Attitudes and preferences of individuals with IBD who have never used medicinal cannabis. You previously indicated you have never used medicinal cannabis. This section will ask about your attitudes towards medicinal cannabis and IBD.

Would you be interested in using medicinal cannabis to treat symptoms relating to your IBD?

- ◯ No
- ◯ Yes, in combination with my current treatments (adjunct)
- ◯ Yes, as a replacement to my current treatments

Why have you not used medicinal cannabis for your IBD to date?

- ☐ It is too expensive
- ☐ Difficulties buying or accessing it
- ☐ Per advice of my health provider (e.g. GP, medical specialist)
- ☐ I am concerned about legal consequences of using an illicit drug
- ☐ I am concerned about on-road random drug testing (RDT)
- ☐ I am worried about how it may interfere with my job/social life
- ☐ Other

(Select all that apply)

If you answered Other, is there another reason you have not used medicinal cannabis for your IBD? ______________________

What is your preference in terms of how you would like to consume medicinal cannabis?	◯ Oral (tablet or capsule)<br>◯ Oral edibles (cake, cookie)<br>◯ Oral liquid (tincture, etc.)<br>◯ Oral spray (mouth or oromucosal)<br>◯ Fresh juice<br>◯ Nasal application (through nose)<br>◯ Rolled into a joint<br>◯ Pipe (plastic, metal, glass)<br>◯ Water pipe/bong<br>◯ Vaporiser<br>◯ Suppository<br>◯ Dabbing or spotting<br>◯ Topical (cream/patch applied to skin)<br>◯ Other<br>(Please only select one option)
If you answered Other, is there another way you would prefer to use medicinal cannabis?	__________________________________

Thank you for being involved!

Where did you first hear about this survey?	◯ Facebook<br>◯ Twitter<br>◯ Other social media<br>◯ Online forum<br>◯ A friend<br>◯ Medicinal cannabis provider<br>◯ Consumer support group<br>◯ Lambert initiative website<br>◯ Doctor/healthcare provider<br>◯ Cannabis Access Clinic<br>◯ Media<br>◯ Other<br>(Please only select one option)
If answered Other, where did you hear about this survey?	__________________________________
Would you be interested in taking part in future clinical trials to test the effects of medical cannabis as a treatment for IBD?	◯ Yes<br>◯ No

If you answered yes and would like a researcher at the University of Sydney to contact you about future clinical trials in medicinal cannabis for inflammatory bowel disease, please click the link below:

https://redcap.sydney.edu.au/surveys/?s=XYPHRD9D3H

Please note that any personal contact details that you provide in the above link will NOT in any way be linked to the main survey so it would be impossible to link your personal information to your survey data.

You have completed 5 of 6 pages so far.

CANNABIS USE FOR IBD SYMPTOMS

Have you ever used cannabis to manage your IBD symptoms?

- ◯ Yes, I currently use cannabis to manage my IBD symptoms
- ◯ Yes, I have previously used cannabis to manage my IBD symptoms but I have now stopped
- ◯ No, I have never used cannabis to manage my IBD symptoms

In your opinion, what should be the legal status of cannabis use in Australia?

- ◯ Cannabis use should be legal for ALL purposes (recreational and medicinal)
- ◯ Cannabis use should be legal for medicinal purposes only
- ◯ Cannabis should be illegal for all purposes
- ◯ Unsure
- ◯ No opinion

PRESENT USERS

Attitudes on medicinal cannabis and use preferences of present users. You indicated you are currently using medicinal cannabis for your IBD. This section will ask about your current use as well as you attitudes and experiences with medicinal cannabis and your IBD.

Which of the following informed your decision to use medicinal cannabis for your IBD?

- ◯ Discovered benefits on my own internet-based media
- ◯ Conventional media
- ◯ Conventional healthcare provider
- ◯ Alternative healthcare practitioner
- ◯ Friend or family member
- ◯ Medicinal cannabis advocacy group
- ◯ Other

How frequently do you use cannabis medicinally to treat symptoms relating to your IBD?

- ◯ Multiple times a day
- ◯ Every day
- ◯ Several times a week
- ◯ Once a week
- ◯ Several times a month
- ◯ Once a month

What is the main way you consume cannabis for medicinal purposes?	◯ Oral (tablet or capsule)<br>◯ Oral edibles (cake, cookie)<br>◯ Oral liquid (oil, tincture)<br>◯ Oral spray (mouth or oromucosal)<br>◯ Fresh juice<br>◯ Nasal application (through nose)<br>◯ Rolled into a joint<br>◯ Pipe (plastic, metal, glass)<br>◯ Water pipe/bong<br>◯ Suppository<br>◯ Dabbing or spotting<br>◯ Topical (cream/patch applied to the skin)<br>◯ Vaporizer<br>◯ Other<br>(Please only select one option)
If you answered Other, which other ways do you consume medicinal cannabis?	__________________________________
What type of medicinal cannabis do you mainly use?	◯ THC only<br>◯ Mainly THC and small amounts of other cannabinoids<br>◯ Approximately equal amounts of THC and CBD<br>◯ Mainly CBD and small amounts of other cannabinoids (e.g. THC)<br>◯ CBD only<br>◯ Unsure/Do not know<br>◯ It varies between batches<br>◯ Other<br>(Please only select one option)
If you answered Other, which other type of cannabis do you mainly use?	__________________________________
What is the main way you access your medicinal cannabis?	◯ Cannabis access clinic<br>◯ I grow my own<br>◯ From friends or family<br>◯ From a medicinal cannabis supplier (not prescribed by a medical practitioner)<br>◯ Prescribed by a medical practitioner and dispensed from a pharmacy<br>◯ From a club or co-operative outlet<br>◯ From an online supplier<br>◯ From an overseas supplier<br>◯ From a recreational dealer<br>◯ Other<br>(Please only select one option)
If you answered Other, which other ways do you access cannabis?	__________________________________
In the last 28 days, have you ever been unable to access medicinal cannabis to treat your IBD?	◯ Yes<br>◯ No
Do you worry about accessing your preferred source of cannabis?	◯ Very much so<br>◯ Somewhat<br>◯ No opinion,<br>◯ Not especially<br>◯ Not at all

Do you consider medicinal cannabis successful in managing your IBD symptoms?	◯ Yes<br>◯ No
Which option best describes how your IBD compares now to how it was before using medicinal cannabis?	◯ Very much better<br>◯ Much better<br>◯ A little better<br>◯ No change<br>◯ A little worse<br>◯ Much worse<br>◯ Very much worse

Which of the following symptoms were altered following medicinal cannabis use and how did this symptom change with medicinal cannabis use?

	Very much better	Much better	A little better	No change	A little worse	Very much worse	I do not have this symptom
Anxiety	◯	◯	◯	◯	◯	◯	◯
Stress	◯	◯	◯	◯	◯	◯	◯
Depression	◯	◯	◯	◯	◯	◯	◯
Memory impairment	◯	◯	◯	◯	◯	◯	◯
Fatigue	◯	◯	◯	◯	◯	◯	◯
Headaches	◯	◯	◯	◯	◯	◯	◯
Reduced appetite	◯	◯	◯	◯	◯	◯	◯
Nausea/vomiting	◯	◯	◯	◯	◯	◯	◯
Diarrhoea	◯	◯	◯	◯	◯	◯	◯
Abdominal pain	◯	◯	◯	◯	◯	◯	◯
Bloating	◯	◯	◯	◯	◯	◯	◯
Cramping	◯	◯	◯	◯	◯	◯	◯
Stool consistency	◯	◯	◯	◯	◯	◯	◯
Stool frequency	◯	◯	◯	◯	◯	◯	◯
Urgency	◯	◯	◯	◯	◯	◯	◯
Obstructive symptoms (fistula, abscess, stricture)	◯	◯	◯	◯	◯	◯	◯
Rectal bleeding	◯	◯	◯	◯	◯	◯	◯
Weight loss	◯	◯	◯	◯	◯	◯	◯
Sleep issues	◯	◯	◯	◯	◯	◯	◯

Were any other symptoms altered following medicinal cannabis use?	◯ Yes<br>◯ No
Describe the other symptoms which improved following medicinal cannabis use	______________________________

What effect did medicinal cannabis have on this symptom?

- ◯ Very much better
- ◯ Much better
- ◯ A little better
- ◯ No change
- ◯ A little worse
- ◯ Much worse
- ◯ Very much worse

Side effects

Indicate whether you have experienced any of the following side effects in the last 12 months when using medicinal cannabis, and, if so, how serious these side effects were.

Note: "mild and tolerable" = symptoms that caused only mind impairment of function and do not require specific treatment (e.g. other medications or stopping medicinal cannabis); "Severe and intolerable" = symptoms that caused impairment of function and/or resulted in either other treatment (e.g. other medications) or resulted in stopping medicinal cannabis use.

	Did not experience	Mild and tolerable	Severe and intolerable
Allergies	◯	◯	◯
Anxiety	◯	◯	◯
Cannabis hyperemesis	◯	◯	◯
Confusion	◯	◯	◯
Constipation	◯	◯	◯
Decreased appetite	◯	◯	◯
Dehydration	◯	◯	◯
Delusions	◯	◯	◯
Depressed mood	◯	◯	◯
Diarrhoea	◯	◯	◯
Dry mouth	◯	◯	◯
Dizziness	◯	◯	◯
Drowsiness or sedation	◯	◯	◯
Eye Irritation	◯	◯	◯
Gastrointestinal irritation	◯	◯	◯
Hallucinations	◯	◯	◯
Headaches	◯	◯	◯
Increased appetite	◯	◯	◯
Lack of energy or fatigue	◯	◯	◯
Memory impairment	◯	◯	◯
Nasopharyngeal complaints	◯	◯	◯
Nausea and/or vomitting	◯	◯	◯
Panic attack	◯	◯	◯

Paranoia	◯	◯	◯
Racing heart/palpitations	◯	◯	◯
Bad taste in mouth	◯	◯	◯
Respiratory complaints	◯	◯	◯
Shaking/tremor/poor movement control	◯	◯	◯
Sleep disturbance	◯	◯	◯
Sweating	◯	◯	◯

Which of the following best describes how your use of each drug changed whilst using medicinal cannabis to treat your symptoms of IBD?

Note: A moderate change = more than 20% difference, a marked change is more than 50% change (e.g. a marked decrease would be reducing the drug by more than half).

	Markedly decreased	Moderately decreased	No change	Moderately increased	Markedly increased	I do not take this medication
Corticosteroids (e.g. prednisone)	◯	◯	◯	◯	◯	◯
Aminosalicylates (e.g. sulfasalazine, mesalamine, olsalazine, balsalazide)	◯	◯	◯	◯	◯	◯
Immunomodulators (e.g. azathioprine, 6-mercaptopurine, tacrolimus, methotrexate)	◯	◯	◯	◯	◯	◯
Calcineurin inhibitors (e.g. cyclosporine, tacrolimus)	◯	◯	◯	◯	◯	◯
Biologic agents (e.g. infliximab, adalimumab, vedolixumab, ustekinumab)	◯	◯	◯	◯	◯	◯
Analgesics (e.g. codeine, oxycodone, tramadol, ibuprofen, Panadol)	◯	◯	◯	◯	◯	◯
Antibiotics (e.g. ciprofloxacin, metronidazole)	◯	◯	◯	◯	◯	◯
Antidepressants/anti-anxiety: (e.g. SSRIs, SNRIs, tricyclics)	◯	◯	◯	◯	◯	◯
Benzodiazepines: (Diazepam, alprazolam)	◯	◯	◯	◯	◯	◯

Are you currently also using cannabis for non-medical purposes (recreationally)?

◯ Yes
◯ No

If yes, how frequently do you use cannabis for non-medical purposes (recreationally)?

◯ Multiple times a day
◯ Every day
◯ Several times a week
◯ Once a week
◯ Several times a month
◯ Once a month

PAST USERS

Attitudes on medicinal cannabis and use preferences of previous but not current users.You previously indicated you have used medicinal cannabis in the past but do not currently use.

This section will ask about your previous use as well as you attitudes and past experiences with medicinal cannabis and your IBD.

Which of the following informed your decision to use medicinal cannabis for your IBD?

- ◯ Discovered benefits on my own Internet-based media
- ◯ Conventional media
- ◯ Conventional healthcare provider
- ◯ Alternative healthcare practitioner
- ◯ Friend or family member
- ◯ Medicinal cannabis advocacy group
- ◯ Other

If chosen Other, please specify:

____

How frequently did you use cannabis medicinally to treat symptoms relating to your IBD?

- ◯ Multiple times a day
- ◯ Every day
- ◯ Several times a week
- ◯ Once a week
- ◯ Several times a month
- ◯ Once a month

What was the main way you consumed cannabis for medicinal purposes?

- ◯ Oral (tablet or capsule)
- ◯ Oral edibles (cake, cookie)
- ◯ Oral liquid (tincture, etc.)
- ◯ Oral spray (mouth or oromucosal)
- ◯ Fresh juice
- ◯ Nasal application (through nose)
- ◯ Rolled into a joint
- ◯ Pipe (plastic, metal, glass)
- ◯ Water pipe/bong
- ◯ Suppository
- ◯ Dabbing or spotting
- ◯ Topical (cream/patch applied to the skin)
- ◯ Vaporizer
- ◯ Other

(Please only select one option)

If you answered Other, which other type of cannabis did you mainly use?

____

What is the main way you accessed your medicinal cannabis?

- ◯ Cannabis access clinic
- ◯ I grow my own
- ◯ From friends or family
- ◯ From a medicinal cannabis supplier (not prescribed by a medical practitioner)
- ◯ Prescribed by a medical practitioner and dispensed from a pharmacy
- ◯ From a club or co-operative outlet
- ◯ From an online supplier
- ◯ From an overseas supplier
- ◯ From a recreational dealer
- ◯ Other

(Please only select one option)

If you answered Other, which other ways did you access cannabis?	__________________________
Have you ever experienced difficulties with accessing medicinal cannabis to treat your IBD?	◯ Yes<br>◯ No
Did you worry about accessing your preferred source of cannabis?	◯ Very much so<br>◯ Somewhat<br>◯ No opinion,<br>◯ Not especially<br>◯ Not at all
Did you consider medicinal cannabis successful in managing your IBD symptoms?	◯ Yes<br>◯ No
Which option best describes how your IBD compares now to how it was before using medicinal cannabis?	◯ Very much better<br>◯ Much better<br>◯ A little better<br>◯ No change<br>◯ A little worse<br>◯ Much worse<br>◯ Very much worse

Which of the following symptoms were altered following medicinal cannabis use and how did this symptom change with medicinal cannabis use?

	Very much better	A little better	No change	A little worse	Much worse	Very much worse	I do not have this symptom
Anxiety	◯	◯	◯	◯	◯	◯	◯
Stress	◯	◯	◯	◯	◯	◯	◯
Depression	◯	◯	◯	◯	◯	◯	◯
Memory impairment	◯	◯	◯	◯	◯	◯	◯
Fatigue	◯	◯	◯	◯	◯	◯	◯
Headaches	◯	◯	◯	◯	◯	◯	◯
Reduced appetite	◯	◯	◯	◯	◯	◯	◯
Nausea/vomiting	◯	◯	◯	◯	◯	◯	◯
Diarrhoea	◯	◯	◯	◯	◯	◯	◯
Abdominal pain	◯	◯	◯	◯	◯	◯	◯
Bloating	◯	◯	◯	◯	◯	◯	◯
Cramping	◯	◯	◯	◯	◯	◯	◯
Stool consistency	◯	◯	◯	◯	◯	◯	◯
Stool frequency	◯	◯	◯	◯	◯	◯	◯
Urgency	◯	◯	◯	◯	◯	◯	◯
Obstructive symptoms (fistula, abscess, stricture)	◯	◯	◯	◯	◯	◯	◯

Rectal bleeding	○	○	○	○	○	○	○
Weight loss	○	○	○	○	○	○	○
Sleep issues	○	○	○	○	○	○	○

Side effects

Indicate whether you experienced any of the following side effects when using medicinal cannabis, and, if so, how serious these side effects were.

Note: "Mild and intolerable" = symptoms that caused only mild impairment of function and do not require specific treatment (e.g. other medications or stopping medicinal cannabis); "Severe or intolerable" = symptoms that caused impairment of function and/or resulted in either other treatment (e.g. other medications) or resulted in stopping medicinal cannabis use.

	I did not experience this side effect	Mild and tolerable	Severe and intolerable
Allergies	○	○	○
Anxiety	○	○	○
Cannabis hyperemesis	○	○	○
Confusion	○	○	○
Constipation	○	○	○
Decreased appetite	○	○	○
Dehydration	○	○	○
Delusions	○	○	○
Depressed mood	○	○	○
Diarrhoea	○	○	○
Dry mouth	○	○	○
Dizziness	○	○	○
Drowsiness or sedation	○	○	○
Eye irritation	○	○	○
Gastrointestinal irritation	○	○	○
Hallucinations	○	○	○
Headaches	○	○	○
Increased appetite	○	○	○
Lack of energy or fatigue	○	○	○
Memory impairment	○	○	○
Nasopharyngeal complaints	○	○	○
Nausea and/or vomiting	○	○	○
Panic attack	○	○	○
Paranoia	○	○	○

Racing heart/palpitations	◯	◯	◯
Bad taste in mouth	◯	◯	◯
Respiratory complaints	◯	◯	◯
Shaking, tremors, poor movement control	◯	◯	◯
Sleep disturbance	◯	◯	◯
Sweating	◯	◯	◯

Why did you discontinue your use of medicinal cannabis for treatment of symptoms relating to your IBD?

- ◯ It was too expensive
- ◯ I had difficulties buying or accessing it
- ◯ Advice of my health provider (e.g. GP, medical specialist)
- ◯ I was concerned about legal consequences of using an illicit drug
- ◯ I was concerned about on-road random drug testing (RDT)
- ◯ My cannabis use interfered with my job/social life
- ◯ Other

If chosen Other, please specify: ______________________________

Would you be interested in using medicinal cannabis in the future to treat symptoms relating to your IBD?

- ◯ Yes
- ◯ No

NEVER USED

Attitudes and preferences of individuals with IBD who have never used medicinal cannabis. You previously indicated you have never used medicinal cannabis. This section will ask about your attitudes towards medicinal cannabis and IBD.

Would you be interested in using medicinal cannabis to treat symptoms relating to your IBD?

- ◯ No
- ◯ Yes, in combination with my current treatments (adjunct)
- ◯ Yes, as a replacement to my current treatments

Why have you not used medicinal cannabis for your IBD to date?

- ☐ It is too expensive
- ☐ Difficulties buying or accessing it
- ☐ Per advice of my health provider (e.g. GP, medical specialist)
- ☐ I am concerned about legal consequences of using an illicit drug
- ☐ I am concerned about on-road random drug testing (RDT)
- ☐ I am worried about how it may interfere with my job/social life
- ☐ Other

(Select all that apply)

If you answered Other, is there another reason you have not used medicinal cannabis for your IBD? ______________________________

What is your preference in terms of how you would like to consume medicinal cannabis?	◯ Oral (tablet or capsule)<br>◯ Oral edibles (cake, cookie)<br>◯ Oral liquid (tincture, etc.)<br>◯ Oral spray (mouth or oromucosal)<br>◯ Fresh juice<br>◯ Nasal application (through nose)<br>◯ Rolled into a joint<br>◯ Pipe (plastic, metal, glass)<br>◯ Water pipe/bong<br>◯ Vaporiser<br>◯ Suppository<br>◯ Dabbing or spotting<br>◯ Topical (cream/patch applied to skin)<br>◯ Other<br>(Please only select one option)
If you answered Other, is there another way you would prefer to use medicinal cannabis?	__________________________________

Thank you for being involved!

Where did you first hear about this survey?	◯ Facebook<br>◯ Twitter<br>◯ Other social media<br>◯ Online forum<br>◯ A friend<br>◯ Medicinal cannabis provider<br>◯ Consumer support group<br>◯ Lambert initiative website<br>◯ Doctor/healthcare provider<br>◯ Cannabis Access Clinic<br>◯ Media<br>◯ Other<br>(Please only select one option)
If answered Other, where did you hear about this survey?	__________________________________
Would you be interested in taking part in future clinical trials to test the effects of medical cannabis as a treatment for IBD?	◯ Yes<br>◯ No

If you answered yes and would like a researcher at the University of Sydney to contact you about future clinical trials in medicinal cannabis for inflammatory bowel disease, please click the link below:

https://redcap.sydney.edu.au/surveys/?s=XYPHRD9D3H

Please note that any personal contact details that you provide in the above link will NOT in any way be linked to the main survey so it would be impossible to link your personal information to your survey data.
