# Supplementary material for: Medicinal Cannabis for Inflammatory Bowel Disease: A Survey of Perspectives, Experiences, and Current Use in Australian Patients
Source: Crohns Colitis 360. 2020 Apr 16;2(2):otaa015. doi: 10.1093/crocol/otaa015 (PMC9802391; doi:10.1093/crocol/otaa015)
Supplement: otaa015_suppl_Supplementary_Material_Advertising [file otaa015_suppl_supplementary_material_advertising.pdf]

Survey Recruitment Materials

Facebook posts on 3 Australian IBD patient forums with their permission:

Crohn's & Colitis Australia
April 29, 2019 ·

Like Page

Researchers at the University of Sydney are seeking people with IBD to complete a survey to investigate patient attitudes towards medicinal cannabis use in Australia.

This is your chance to share your experiences and perspectives on cannabis as a treatment for the gastrointestinal symptoms of IBD.

Participating in this survey will provide data that may inform future clinical trials of cannabis-based therapies for people living with IBD.

For further information or to complete the survey click here:
bit.ly/SydUSurvey

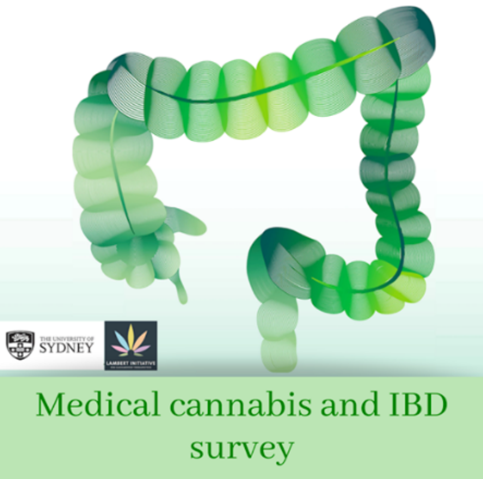

288 · 288 Comments 94 Shares

Lambert Initiative for Cannabinoid Therapeutics ▶ Bowel Cancer Australia
May 14, 2019 ·

Like Page

Do you have Inflammatory Bowel Disease (IBD) or know someone with IBD?

Clinical researchers at the University of Sydney have launched a survey exploring IBD patient experiences and attitudes towards the use of cannabis to manage symptoms of IBD.

If you have an IBD diagnosis, are 18 years of age or older and receive healthcare in Australia, we'd like to hear from you. The survey will be confidential, take 15-20 minutes of your time and the results will inform future clinical trials of cannabis-based therapies.

Follow this link to participate: https://redcap.sydney.edu.au/surveys/?s=XCTA7C8XJC

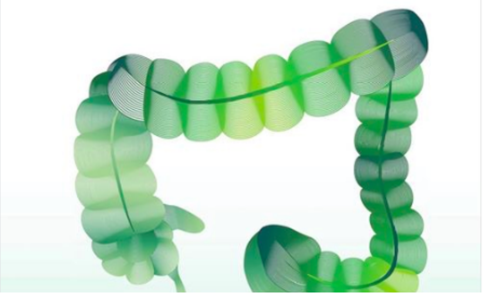

The Gut Foundation
May 7, 2019 ·

Do you have or know someone with IBD?
Clinical researchers at the University of Sydney have launched a survey exploring IBD patient experiences and attitudes towards the use of cannabis as a way to manage symptoms.
Participate by visiting our website below.

GUTFOUNDATION.COM.AU
Treatment and prevention of gastrointestinal disease and conditions
Guidance on conditions and diseases including bowel cancer, irritable bowel syndrome, bloating, reflux, constipation, diarrhoea (Diarrhea)

University of Sydney media story:

THE UNIVERSITY OF SYDNEY | Study | Campus life | Research | Engage with us | About us | News & opinion

← Home
← News & opinion
News
2020: all news
Arts & culture
Business & economics
Campus & community
Government & politics
Health & medicine
Law & society
Science & technology
Subscribe
Podcasts
Videos
News archive
Media contacts
Find an expert

News_

Cannabis use in inflammatory bowel disease: new surveys announced

18 April 2019

The first national surveys of patients and specialists across Australia

Two new surveys announced for patients using or considering using medicinal cannabis to ease the symptoms of inflammatory bowel disease and for specialists caring for these patients.

The anonymous survey will ask patients how their Inflammatory Bowel Disease symptoms affect their daily lives, including work and productivity, their adherence to their current medications and their attitudes on medicinal cannabis and use preferences.

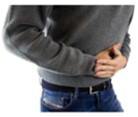

Inflammatory Bowel Disease (IBD) is a debilitating condition.

Inflammatory Bowel Disease (IBD) is an umbrella term describing chronic and relapsing inflammatory diseases of the gastrointestinal tract, including ulcerative colitis and Crohn's disease.

Affected individuals experience abdominal pain, weight loss, diarrhoea and bleeding with a high risk of developing colorectal and small bowel cancers. It is a debilitating condition that often significantly affects a patient's day to day quality of life, which is why some are turning to cannabis for symptom relief.

In 2016, clinical researchers at The University of Sydney's Lambert Initiative for Cannabinoid Therapeutics launched the Cannabis as Medicine Survey, 2016, surveying 1,749 Australians who reported using cannabis for medicinal purposes.

Of those surveyed, over 200 people reported using cannabis to manage symptoms of Inflammatory Bowel Disease (IBD).

The newest Cannabis as Medicine Survey (2018), which closed earlier this year, is being analysed for publication and shows data consistent with the 2016 finding. This issue is not going away.

Patients report dissatisfaction managing their IBD with available treatment options. Anecdotal reports of cannabis improving symptoms of IBD paired with increased interest from the clinical and patient communities has prompted the Lambert Initiative to focus their attention on cannabis use specifically for IBD. Two surveys are now active and independently explore patient and specialist perspectives. This approach hopes to get a snapshot of Australian use and attitudes from both sides of the story.

"Medicinal cannabis is an important issue that is popping up in our clinics and we need to know how our patients may be using it to address their IBD symptoms. The patient survey will help to understand this," says Dr Crispin Corte, specialist gastroenterologist and clinical lead of IBD at Royal Prince Alfred Hospital, and one of the investigators on this study.

The specialist survey follows in the wake of the Lambert Initiative's highly cited cross-sectional survey of 640 Australian general practitioners attitudes toward medicinal cannabis run in collaboration with HealthEd, a learning resource for GPs, last year.

Do you have IBD or know somebody who does? The IBD patient survey is now active and can be completed online.

Share

Want to participate?
Sign up for the IBD patient survey →

Learn more
About medicinal cannabis →

Elissa Blake
Assistant Media Adviser (Science)
Phone
+61 2 8627 9053
Mobile
+61 408 565 604
Email
elissa.blake@sydney.edu.au

Related Articles

News_ 27 March 2019

News_ 10 March 2017

News_ 05 September 2018

Twitter Advertisement:

Lambert Initiative
@Lambert_Usyd

Have you been diagnosed with #inflammatoryboweldisease? Are you an #Australian aged 18+? Researchers at @Lambert_Usyd have launched an anonymous online #survey on #IBD patient experiences of #cannabis to manage their symptoms - complete survey here redcap.sydney.edu.au/surveys/?s=XCT...

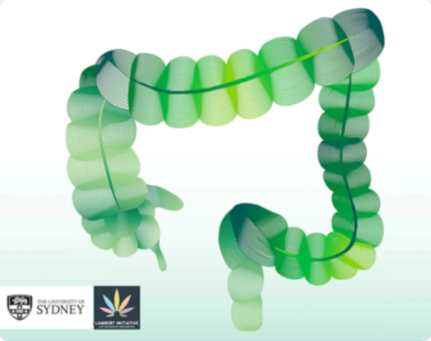

6:08 PM · Apr 18, 2019 · Twitter Web Client

Survey Recruitment Materials cont.

Website posts on 2 Australian IBD patient forums:

Not secure | gutfoundation.com.au/blog/USYD-IBD-management-cannabis-study?fbclid=IwAR3-5_46iUo3GF9HDEEuPkBlk-B6s2SAdH5Kn-zKfnZbTE6mRD_Xh4ckH...

GUT FOUNDATION BLOG

DO YOU HAVE INFLAMMATORY BOWEL DISEASE (IBD) OR KNOW SOMEONE WITH IBD?

GutFoundation Admin - Monday, April 29, 2019

Do you have Inflammatory Bowel Disease (IBD) or know someone with IBD?

Clinical researchers at the University of Sydney have launched a survey exploring IBD patient experiences and attitudes towards the use of cannabis as a way to manage symptoms of IBD . Participating in this survey will provide data that may inform future clinical trials of cannabis-based therapies for people living with IBD.

University of Sydney Human Research Ethics Committee (Project ID: 2018/989). Research led by Professor Iain McGregor, a leader in the medicinal cannabis field alongside a Sydney-based team of academic researchers including several IBD specialist clinicians.

To participate click here.

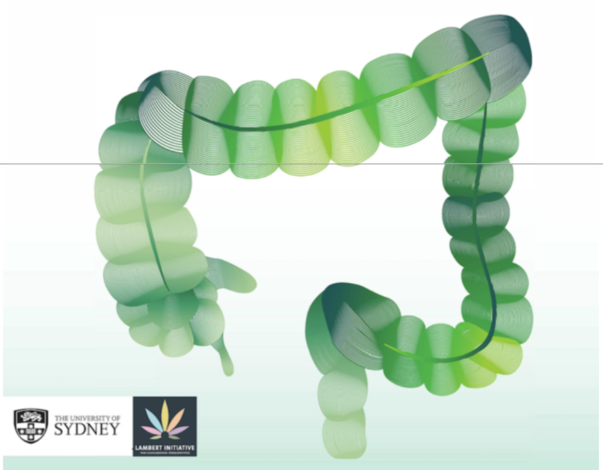

For more information on IBD you can purchase a copy of The Gut Foundations *Inflammatory Bowel Disease* Pamphlet.

Recent Posts

- Health Message from Professor Terry Bolin
- Do you have Inflammatory Bowel Disease (IBD) or know someone with IBD?
- Launch of the Good Gut Cookbook
- Queen's Birthday Honour for our President and Founder, Professor Terry Bolin
- IBD Awareness 2017
- IBS Campaign Wrap
- IBS Awareness Month - Amy's Story
- IBS Awareness Month - Rafaella's Story
- Irritable Bowel Syndrome Info
- Inflammatory Bowel Disease Infographic

Tags

Books IBD Pamphlets IBS

- Books (1)
- IBD (2)
- IBS (4)
- Pamphlets (1)

Archive

- June 2019 (1)
- April 2019 (1)

crohnsandcolitis.com.au/research/clinical-trials/current-researc/

Crohn's&Colitis™ AUSTRALIA *making life more liveable*

Search Site

REGISTER | LOGIN

HELPLINE: 1800 138 029

CC HUB ONLINE SUPPORT

HOME • CROHN'S & COLITIS • MEMBERSHIP • SUPPORT • HOW TO HELP • RESEARCH & ADVOCACY • ABOUT Donate

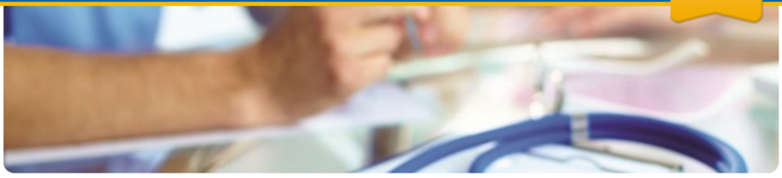

Crohn's & Colitis Australia > Research > Clinical Trials > Current research

CURRENT RESEARCH

USE OF CANNABIS IN MANAGING SYMPTOMS OF IBD

Clinical researchers at the University of Sydney have launched a survey exploring IBD patient experiences and attitudes towards the use of cannabis as a way to manage symptoms of IBD.

We are looking for participants who have an IBD diagnosis, are 18 years of age or older and receive healthcare in Australia.

With a rising interest in alternative treatments for IBD, we hope to shed light upon rate of use, perceived effectiveness and attitudes towards use of cannabis for gastrointestinal symptoms in IBD patients in Australia. Participating in this survey will provide data that may inform future clinical trials of cannabis-based therapies for people living with IBD.

Your participation in the survey is completely voluntary, confidential and you may stop at any time. It is expected that the survey will take approximately 15-20 minutes of your time. This study has been approved by the University of Sydney Human Research Ethics Committee (Project ID: 2018/989).

Research will be led by Professor Iain McGregor, a leader in the medicinal cannabis field, alongside a Sydney-based team of academic researchers including several IBD specialist clinicians.

The survey and further information are **available here**.
